# Supplementary material for: Clinical and molecular characterization of virus-positive and virus-negative Merkel cell carcinoma
Source: Genome Med. 2020 Mar 18;12:30. doi: 10.1186/s13073-020-00727-4 (PMC7081548; doi:10.1186/s13073-020-00727-4)
Supplement: Supplementary file 2 — Additional file 2: Fig. S1. Oncoprint for all genes in this study. Oncoprint summarizing point mutations and CNVs for all genes and patients in this study. Sample are in order of descending TMB and genes are in order of highest point mutations to least. Fig. S2. Network graph for recurrent CNVs. Network graph with all significantly (q < 0.05) co-occurrent CNVs showing how they cluster into reoccurring groups. Fig. S3. Additional file 4.pdf: CNV frequency by cluster for all patients. Cluster number is shown in gray bars above the bar plots representing amplifications/gains (red) and deletions/losses (blue). Below the bar plots is a heat map of all CNVs (genes, x-axis) (amplifications/gains, red; deletions/losses, blue; no change, gray) across all samples (y-axis) annotated by cluster and chromosome. On the left side pRB, p53 shown in gray and black for 1 or 2 copy loss/mutant, respectively. Presence of UV mutations are shown in black. Fig. S4. RFS survival divided by CNV cluster 5. Kaplan-meier plot for MCC patients split by presence or absence of CNV cluster 5 showing no difference in regression free survival (RFS). Fig. S5: Plot of tumor sampling by patient for sequencing. Plot of all patients along the y axis and time since diagnosis on the x axis. Initial diagnosis, primary biopsy sequencing, recurrence biopsy sequencing, death, and last contact times are plotted as applicable. [file 13073_2020_727_MOESM2_ESM.pdf]

Figure S1

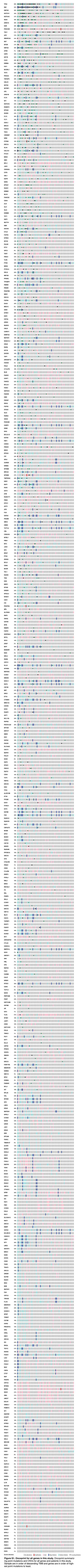

Figure S1. Oncoprint for all genes in this study. Oncoprint summarizing point mutations and CNVs for all genes and patients in this study. Sample are in order of descending TMB and genes are in order of highest point mutations to least.

Figure S2

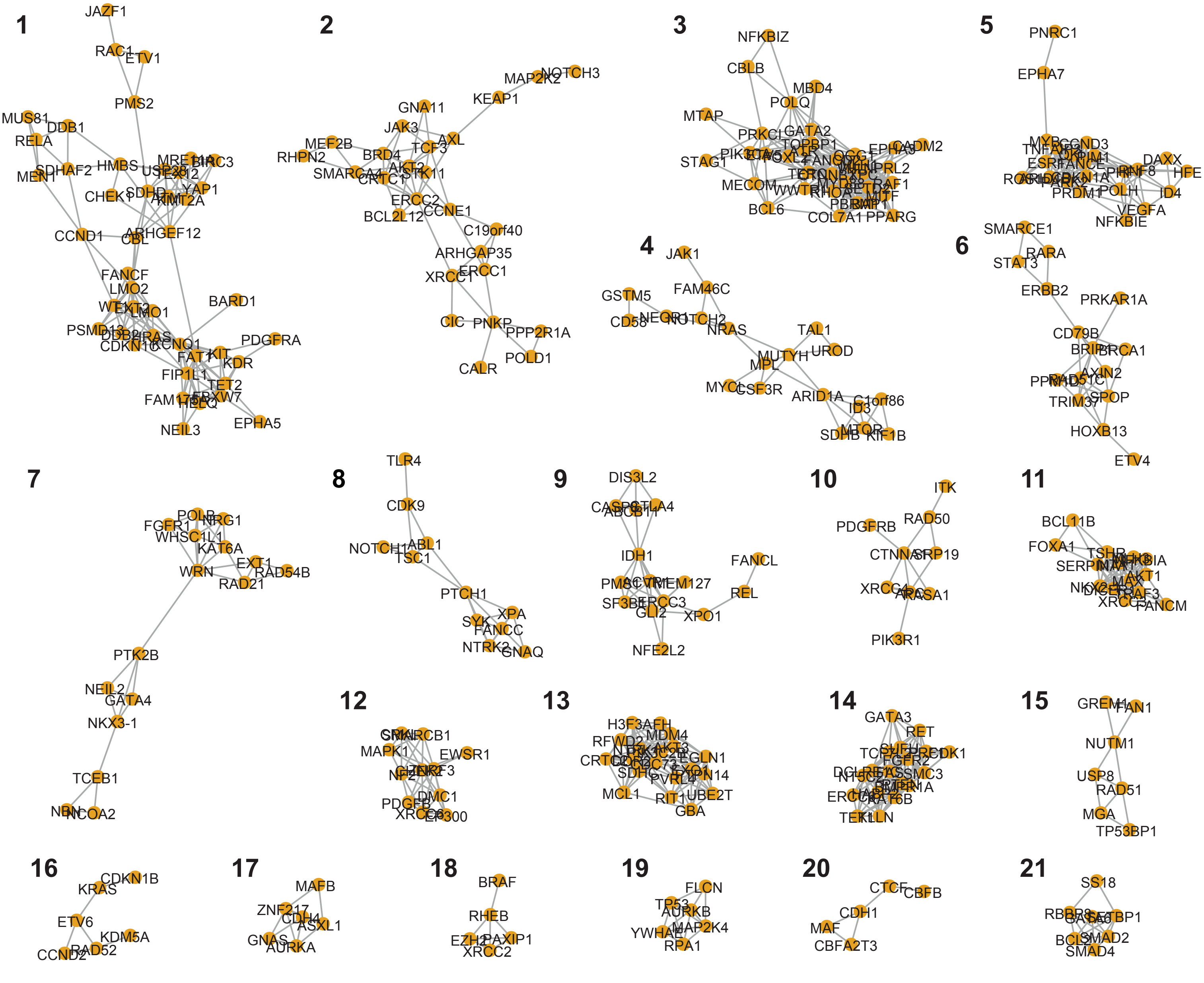

Figure S2. Network graph for recurrent CNVs. Network graph with all significantly (q<0.05) co-occurrent CNVs showing how they cluster into reoccurring groups.

Figure S3

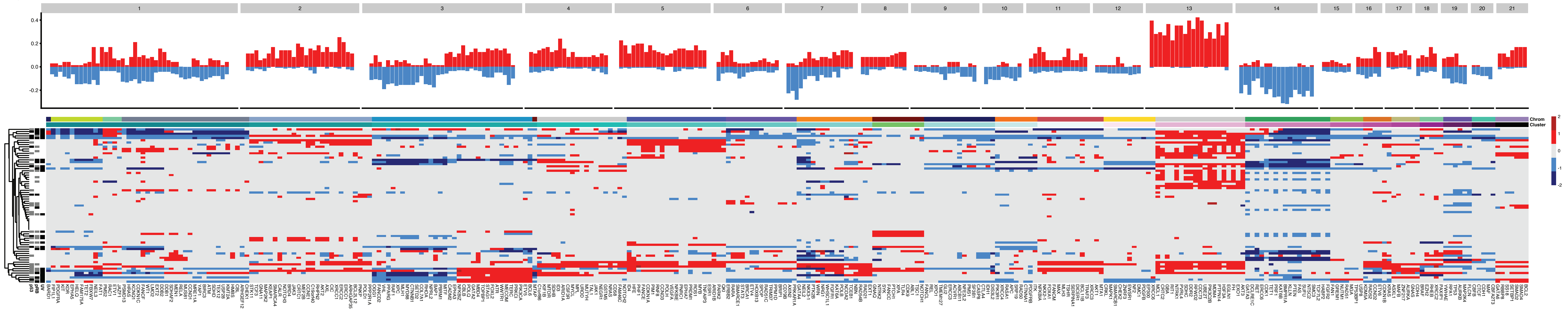

Figure S3. CNV frequency by cluster for all patients. Summary of all genes and CNV clusters in all patients in this cohort. Cluster number is shown in grey bars above the bar plots representing amplifications/gains (red) and deletions/losses (blue). Below the bar plots is a heat map of all CNVs (genes, x-axis) (amplifications/gains, red; deletions/losses, blue; no change, grey) across all samples (y-axis) annotated by cluster and chromosome. On the left side pRB, p53 shown in grey and black for 1 or 2 copy loss/mutant, respectively. Presence of UV mutations are shown in black.

Figure S4

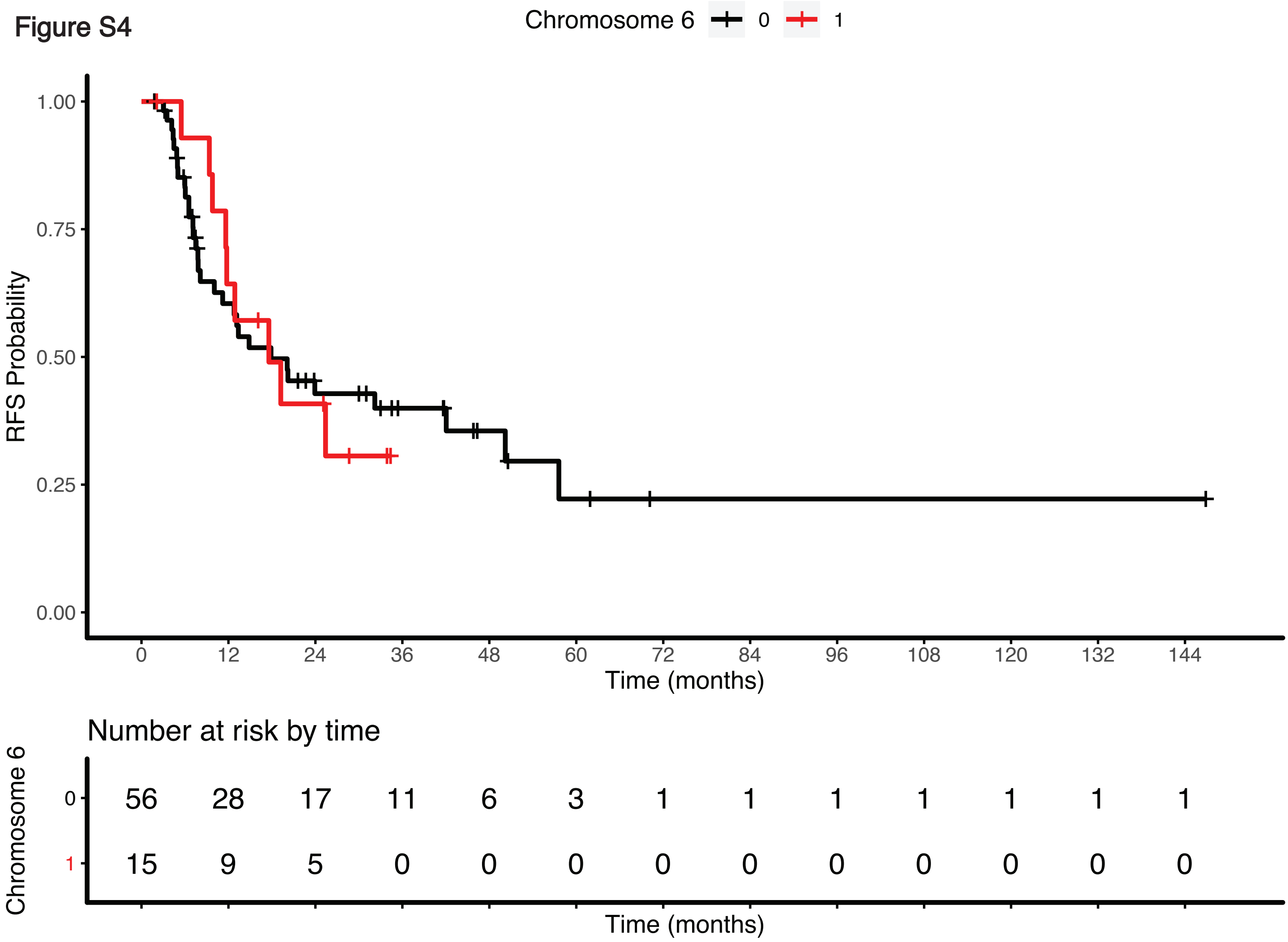

Figure S4. RFS survival divided by CNV cluster 5. Kaplan-meier plot for MCC patients split by presence or absence of CNV cluster 5 showing no difference in regression free survival (RFS)

Figure S5

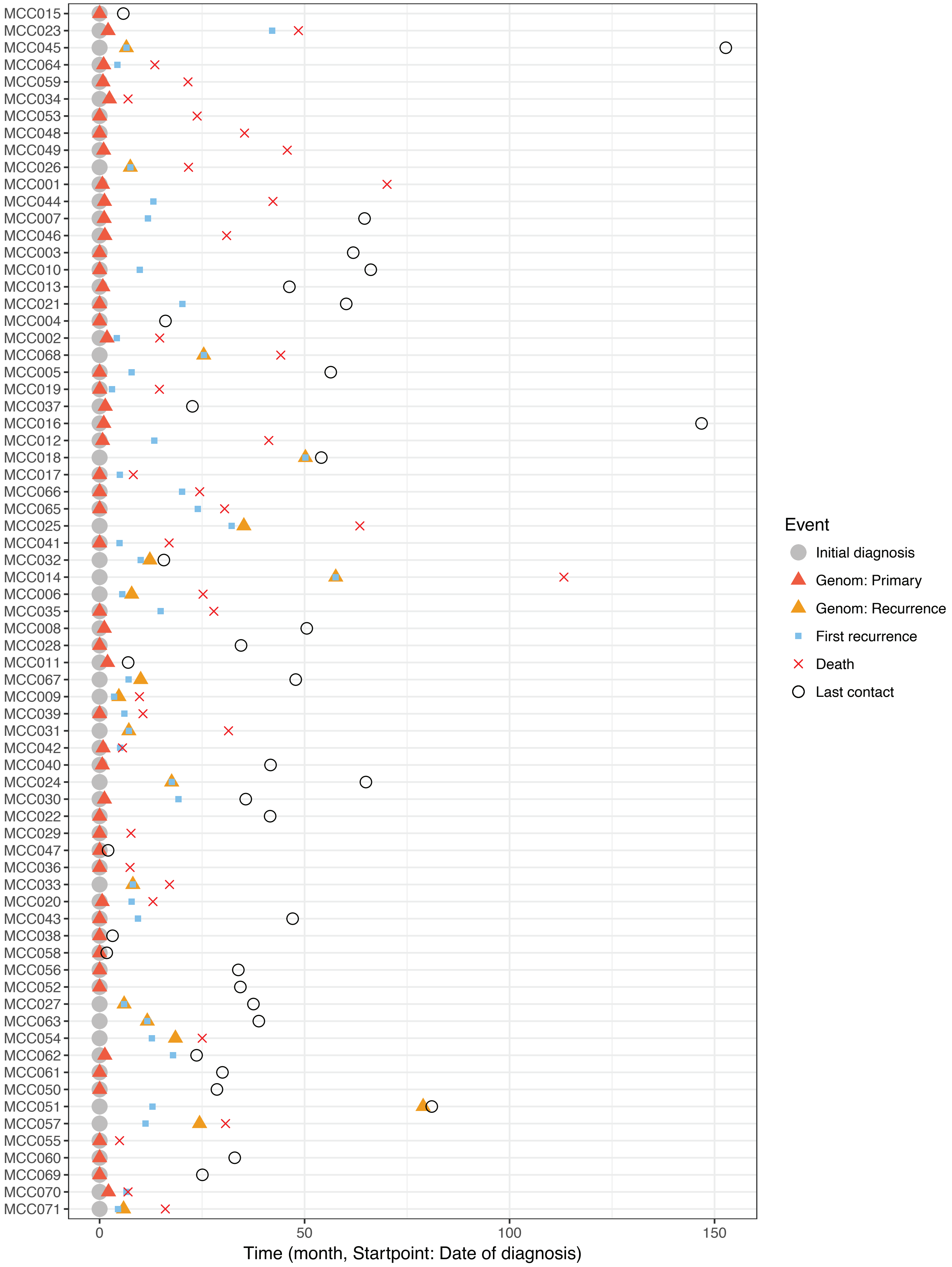

Figure S5. Plot of tumor sampling by patient for sequencing. Plot of all patients along the y axis and time since diagnosis on the x axis. Initial diagnosis, primary biopsy sequencing, recurrence biopsy sequencing, death, and last contact times are plotted as applicable.
