# Supplementary material for: Clinical and molecular characterization of virus-positive and virus-negative Merkel cell carcinoma
Source: Genome Med. 2020 Mar 18;12:30. doi: 10.1186/s13073-020-00727-4 (PMC7081548; doi:10.1186/s13073-020-00727-4)
Supplement: Supplementary file 3 — Additional file 3: Fig. S6. Assembly graphs for all integration events. 28 assembly graphs annotated by MCPyV genome position (colors labeling each segment are under the header “as.factor(V9)”). The “V3” variable represents the coverage of each contig as determined by SPAdes and contigs are scaled to reflect this value. [file 13073_2020_727_MOESM3_ESM.pdf]

Figure S6. Assembly graphs for all integration events. 28 assembly graphs annotated by MCPyV genome position (colors labeling each segment are under the header “as.factor(V9)”). The “V3” variable represents the coverage of each contig as determined by SPAdes and contigs are scaled to reflect this value.

MCC001.pdf

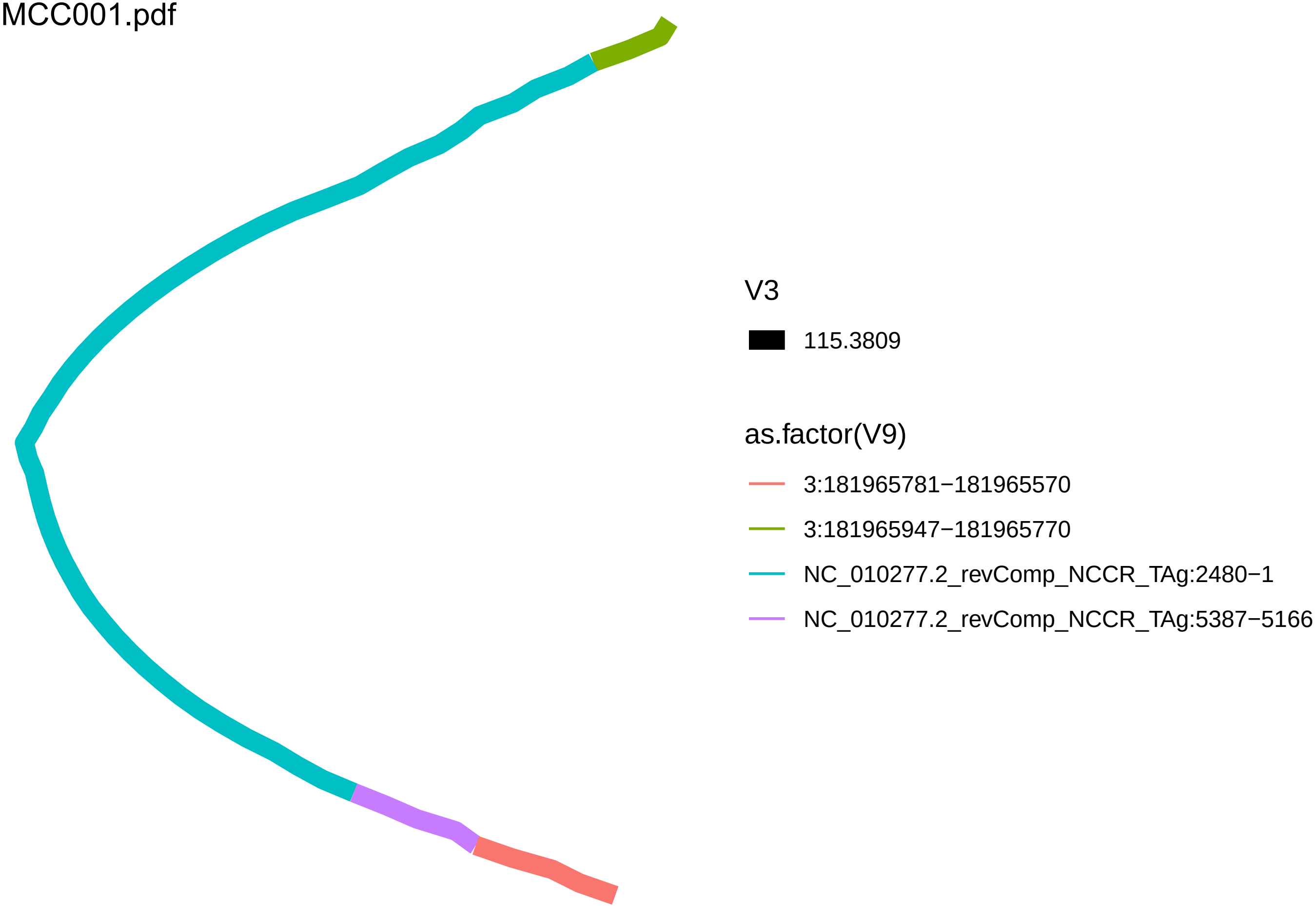

V3
115.3809
as.factor(V9)
3:181965781−181965570
3:181965947−181965770
NC_010277.2_revComp_NCCR_TAg:2480−1
NC_010277.2_revComp_NCCR_TAg:5387−5166

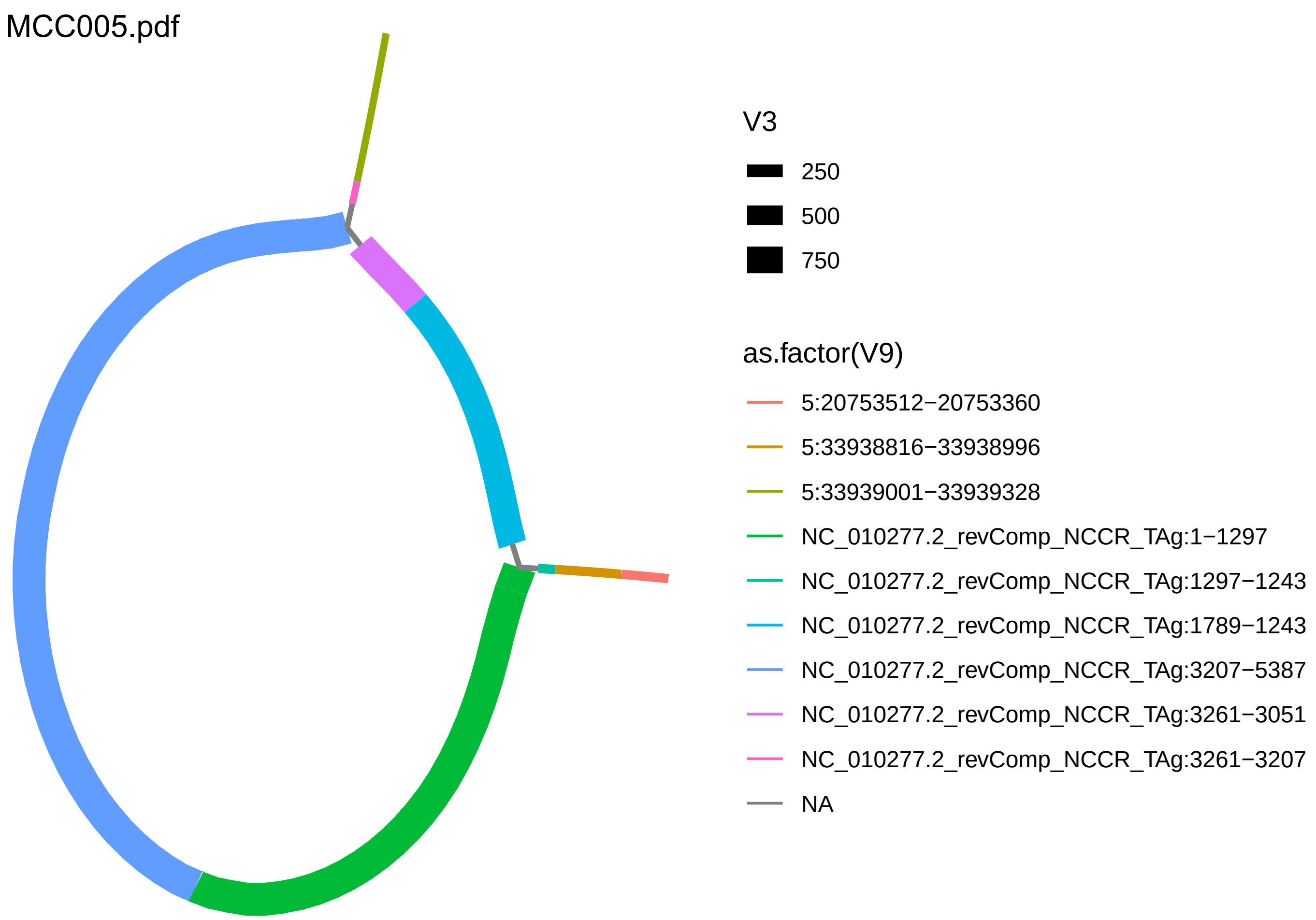
MCC005.pdf
V3
250
500
750
as.factor(V9)
5:20753512−20753360
5:33938816−33938996
5:33939001−33939328
NC_010277.2_revComp_NCCR_TAg:1−1297
NC_010277.2_revComp_NCCR_TAg:1297−1243
NC_010277.2_revComp_NCCR_TAg:1789−1243
NC_010277.2_revComp_NCCR_TAg:3207−5387
NC_010277.2_revComp_NCCR_TAg:3261−3051
NC_010277.2_revComp_NCCR_TAg:3261−3207
NA

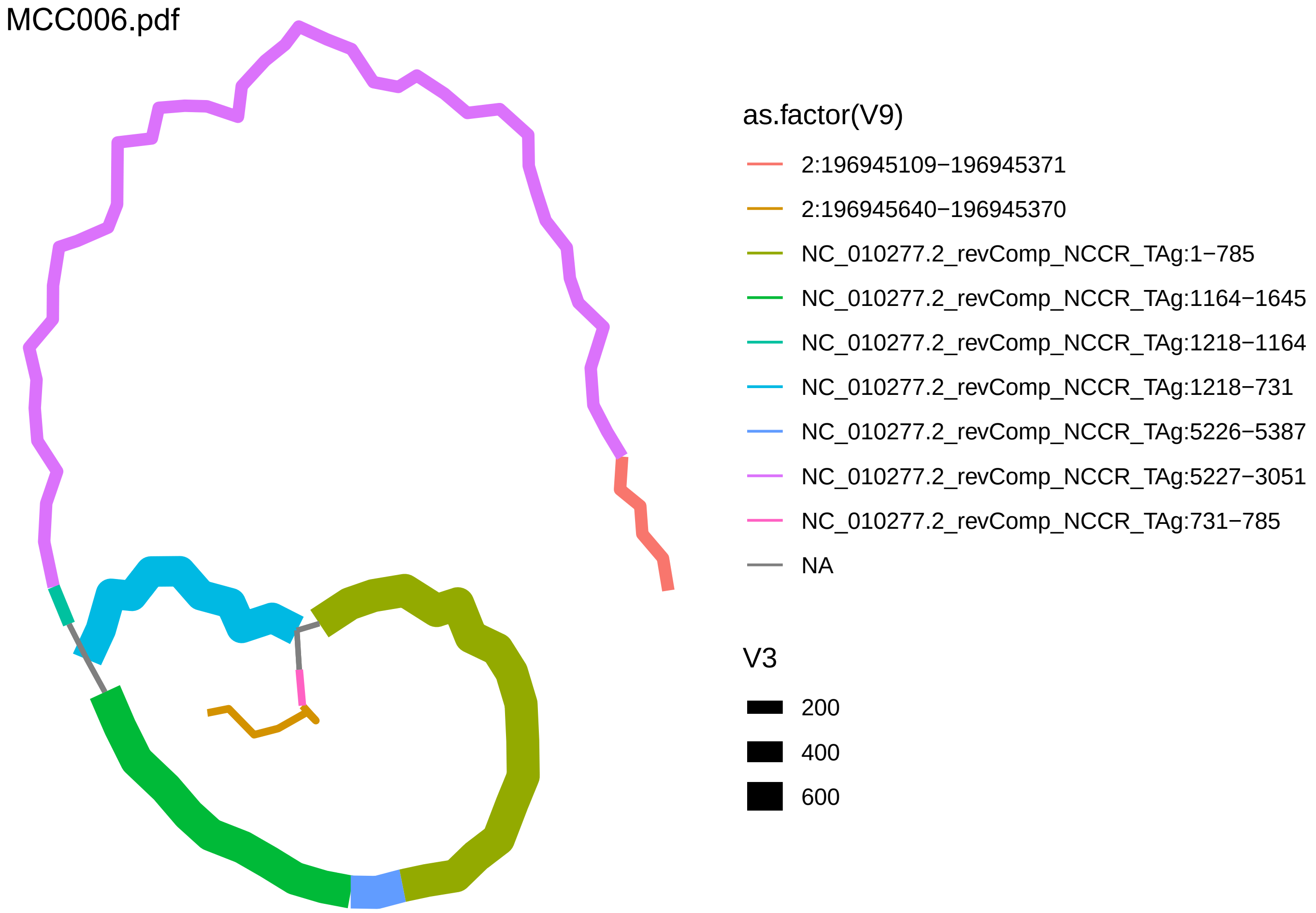
MCC006.pdf
as.factor(V9)
2:196945109−196945371
2:196945640−196945370
NC_010277.2_revComp_NCCR_TAg:1−785
NC_010277.2_revComp_NCCR_TAg:1164−1645
NC_010277.2_revComp_NCCR_TAg:1218−1164
NC_010277.2_revComp_NCCR_TAg:1218−731
NC_010277.2_revComp_NCCR_TAg:5226−5387
NC_010277.2_revComp_NCCR_TAg:5227−3051
NC_010277.2_revComp_NCCR_TAg:731−785
NA
V3
200
400
600

MCC008.pdf

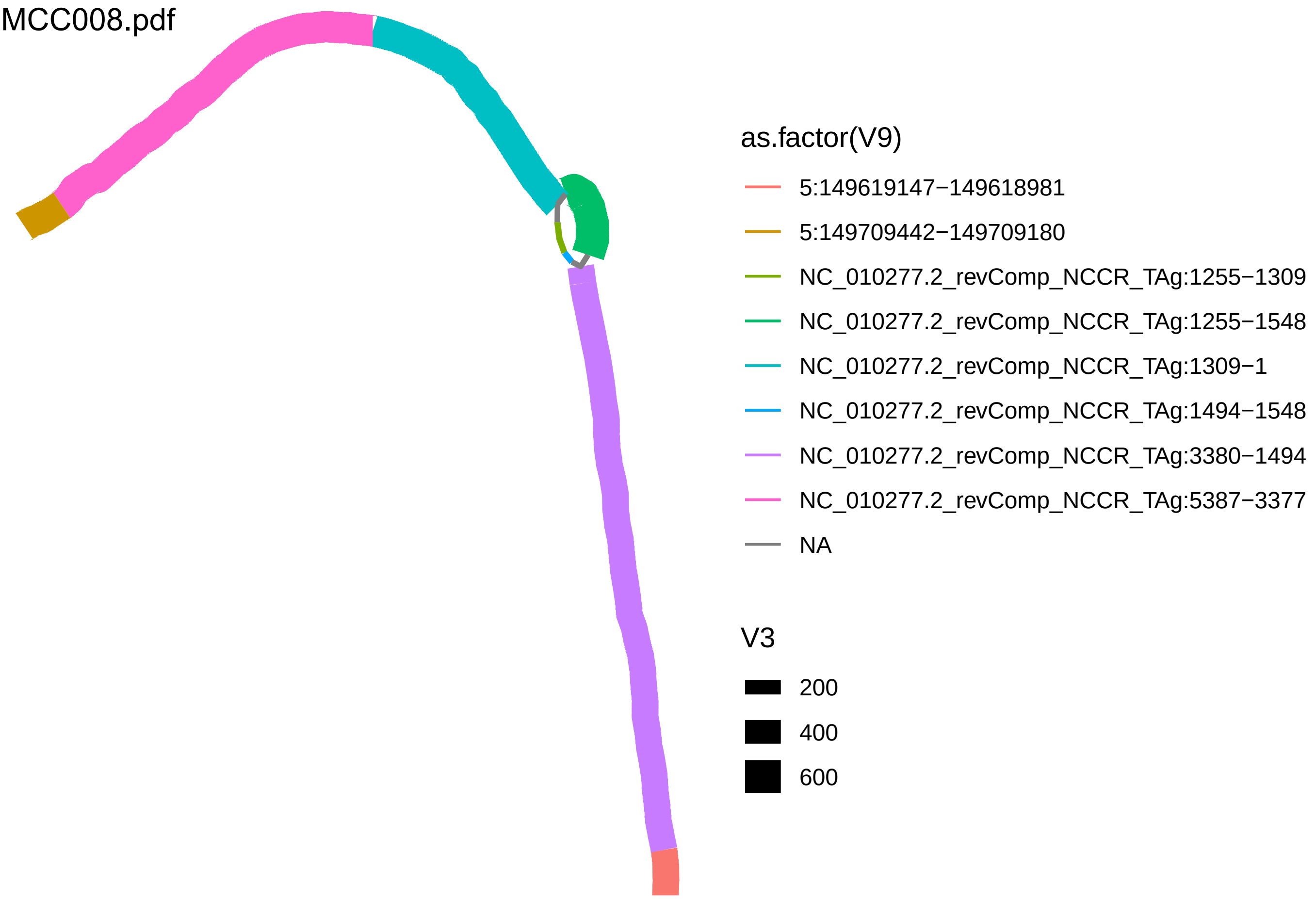

as.factor(V9)
5:149619147−149618981
5:149709442−149709180
NC_010277.2_revComp_NCCR_TAg:1255−1309
NC_010277.2_revComp_NCCR_TAg:1255−1548
NC_010277.2_revComp_NCCR_TAg:1309−1
NC_010277.2_revComp_NCCR_TAg:1494−1548
NC_010277.2_revComp_NCCR_TAg:3380−1494
NC_010277.2_revComp_NCCR_TAg:5387−3377
NA
V3
200
400
600

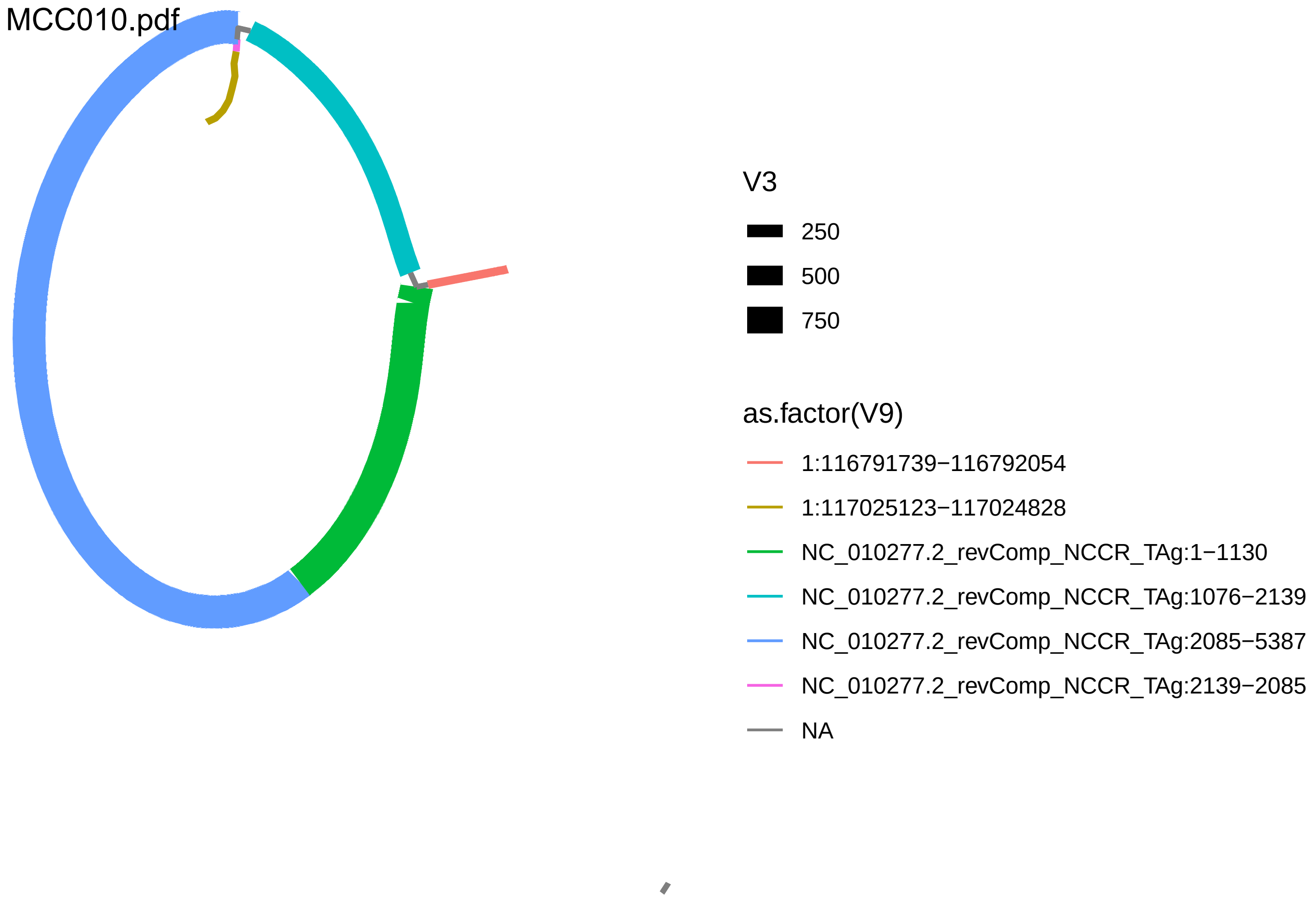
MCC010.pdf
V3
250
500
750
as.factor(V9)
1:116791739−116792054
1:117025123−117024828
NC_010277.2_revComp_NCCR_TAg:1−1130
NC_010277.2_revComp_NCCR_TAg:1076−2139
NC_010277.2_revComp_NCCR_TAg:2085−5387
NC_010277.2_revComp_NCCR_TAg:2139−2085
NA

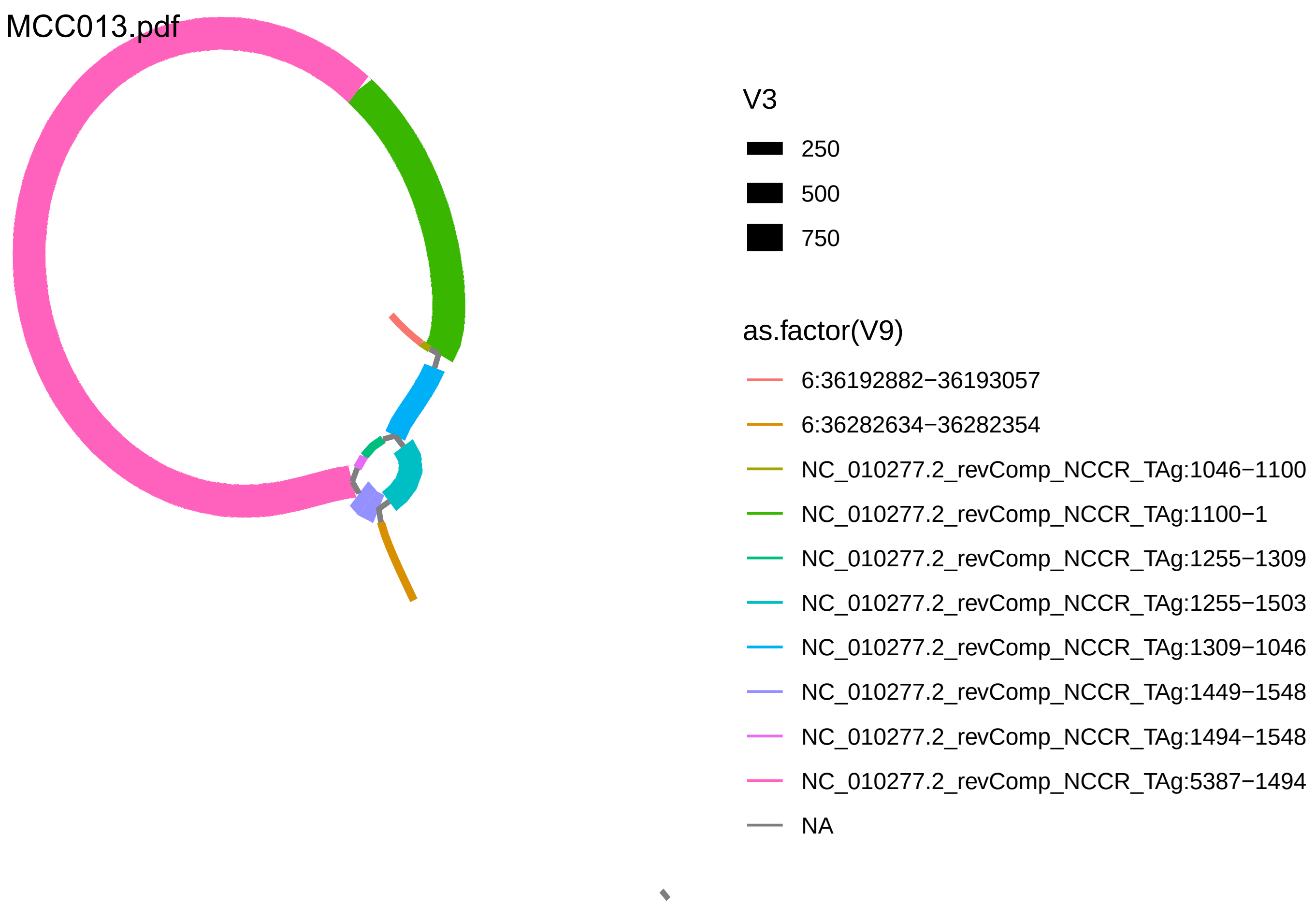

MCC013.pdf
V3
250
500
750
as.factor(V9)
6:36192882−36193057
6:36282634−36282354
NC_010277.2_revComp_NCCR_TAg:1046−1100
NC_010277.2_revComp_NCCR_TAg:1100−1
NC_010277.2_revComp_NCCR_TAg:1255−1309
NC_010277.2_revComp_NCCR_TAg:1255−1503
NC_010277.2_revComp_NCCR_TAg:1309−1046
NC_010277.2_revComp_NCCR_TAg:1449−1548
NC_010277.2_revComp_NCCR_TAg:1494−1548
NC_010277.2_revComp_NCCR_TAg:5387−1494
NA

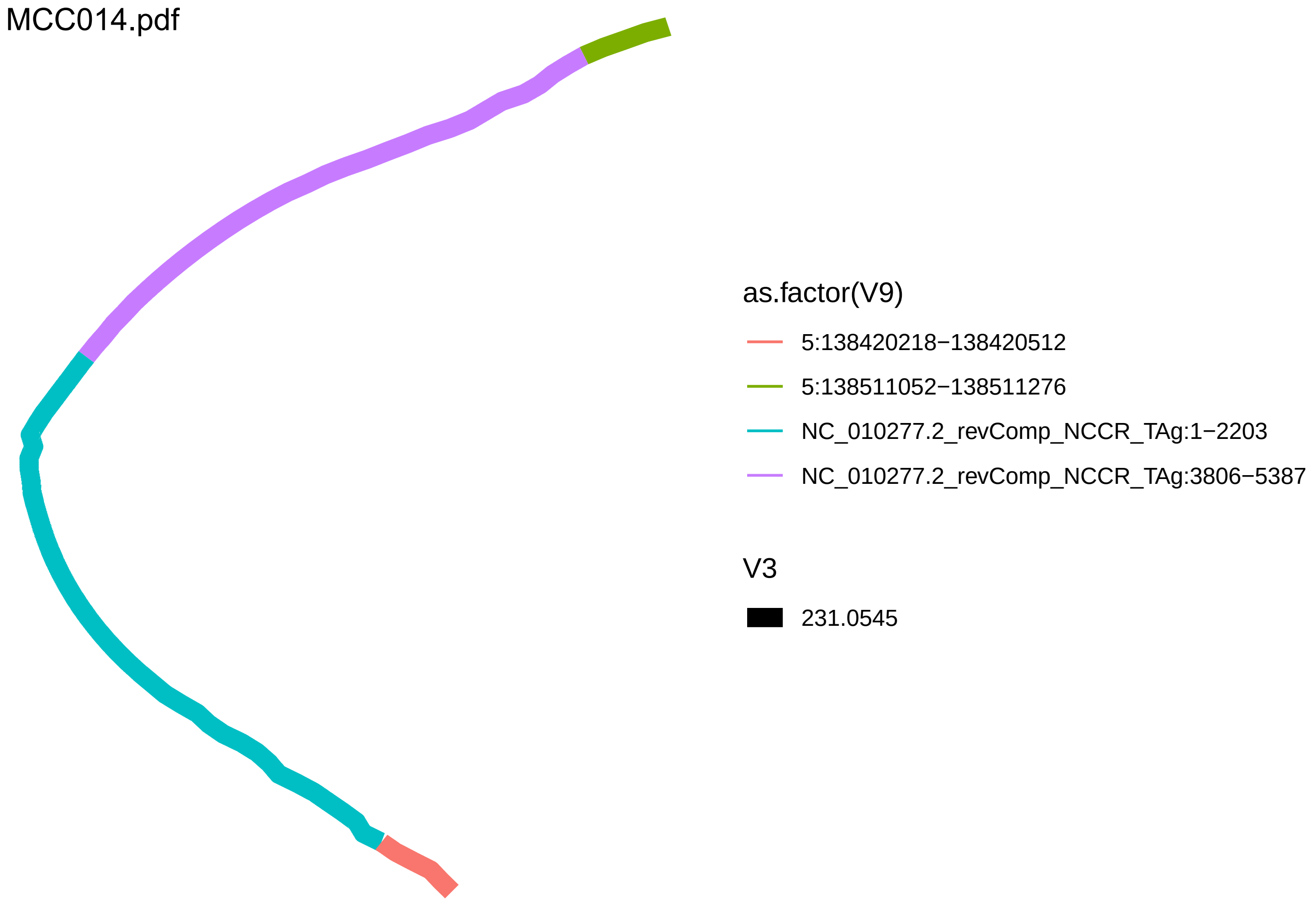

MCC014.pdf
as.factor(V9)
5:138420218−138420512
5:138511052−138511276
NC_010277.2_revComp_NCCR_TAg:1−2203
NC_010277.2_revComp_NCCR_TAg:3806−5387
V3
231.0545

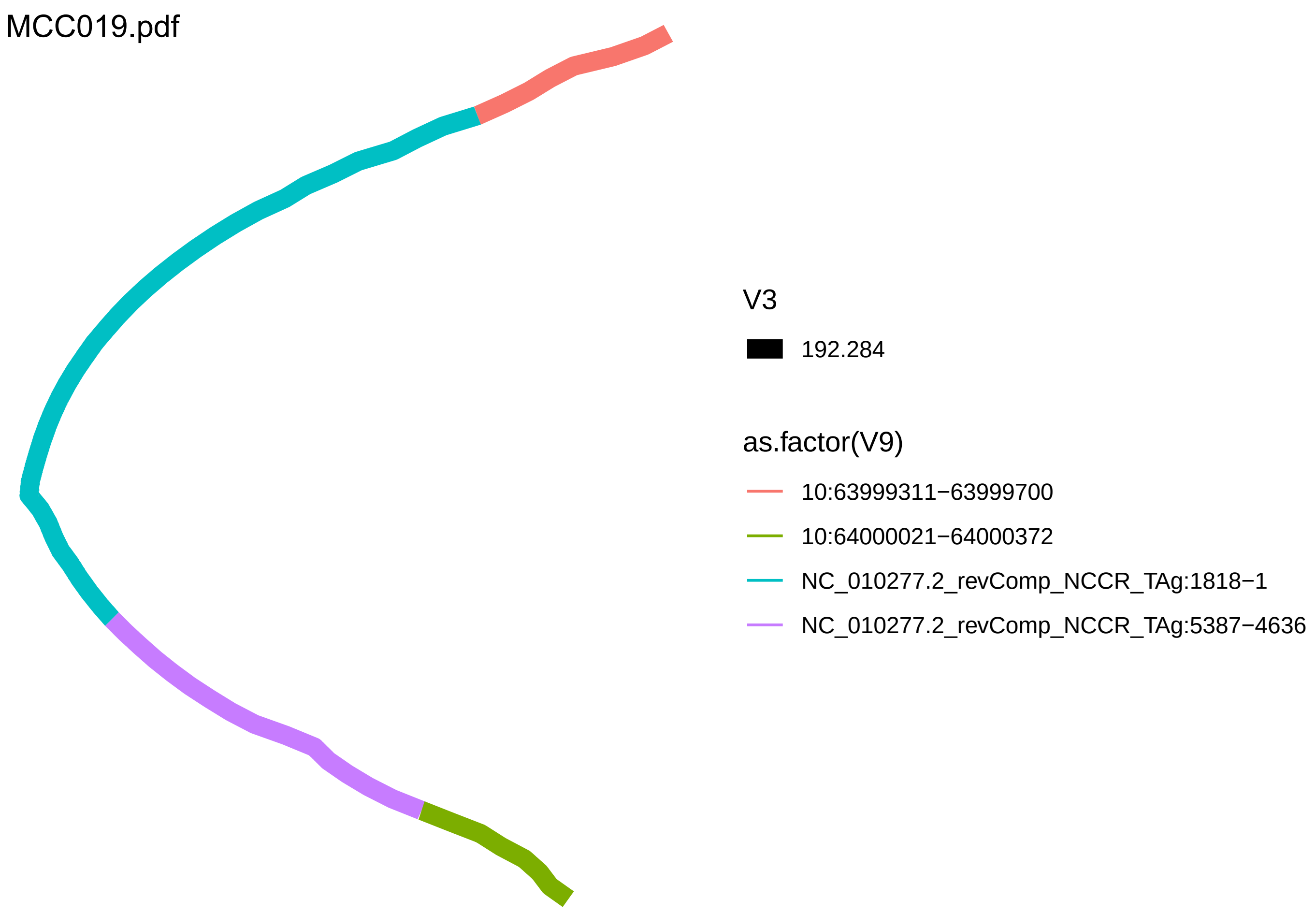

MCC019.pdf
V3
192.284
as.factor(V9)
10:63999311−63999700
10:64000021−64000372
NC_010277.2_revComp_NCCR_TAg:1818−1
NC_010277.2_revComp_NCCR_TAg:5387−4636

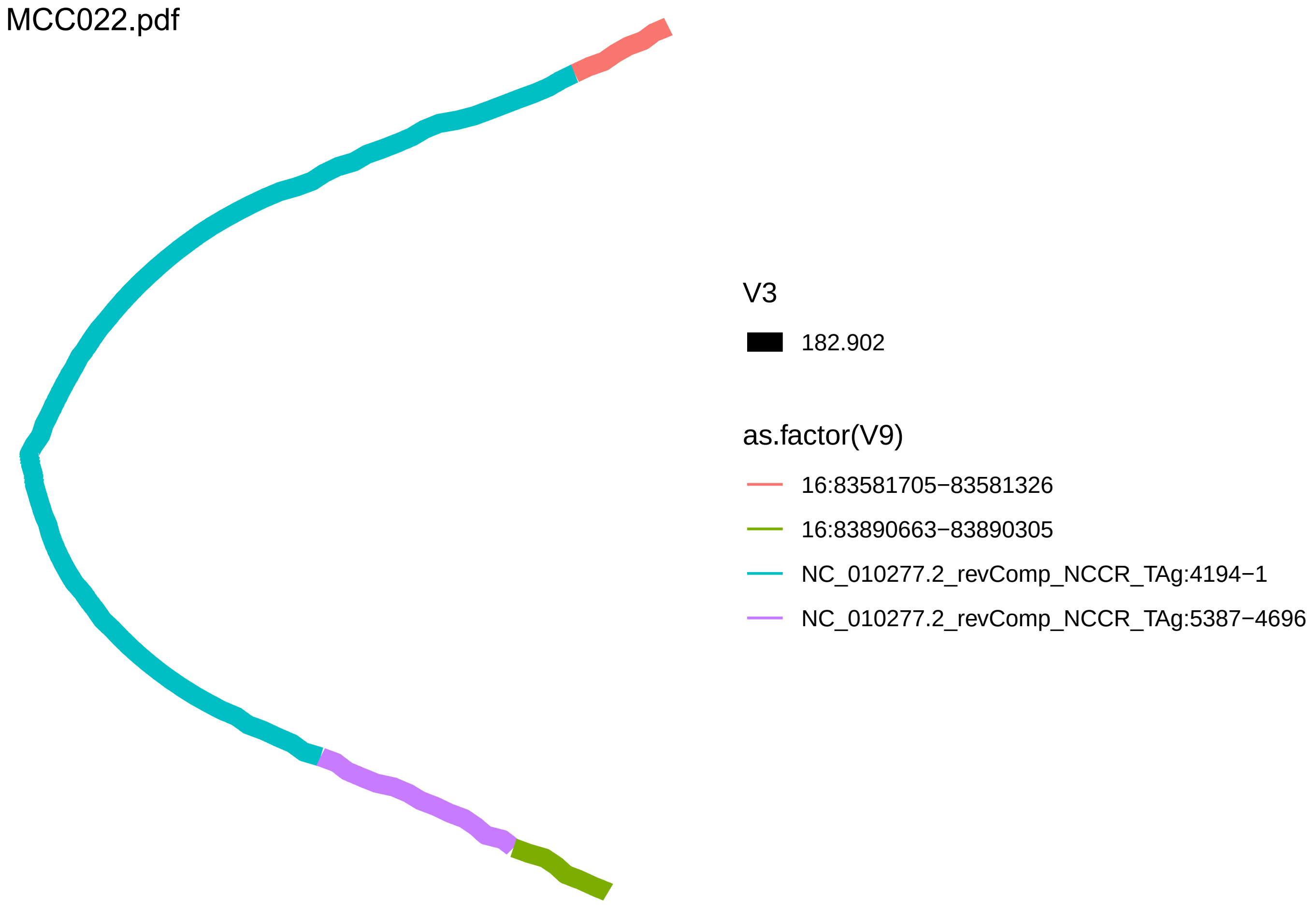
MCC022.pdf
V3
182.902
as.factor(V9)
16:83581705−83581326
16:83890663−83890305
NC_010277.2_revComp_NCCR_TAg:4194−1
NC_010277.2_revComp_NCCR_TAg:5387−4696

MCC024.pdf

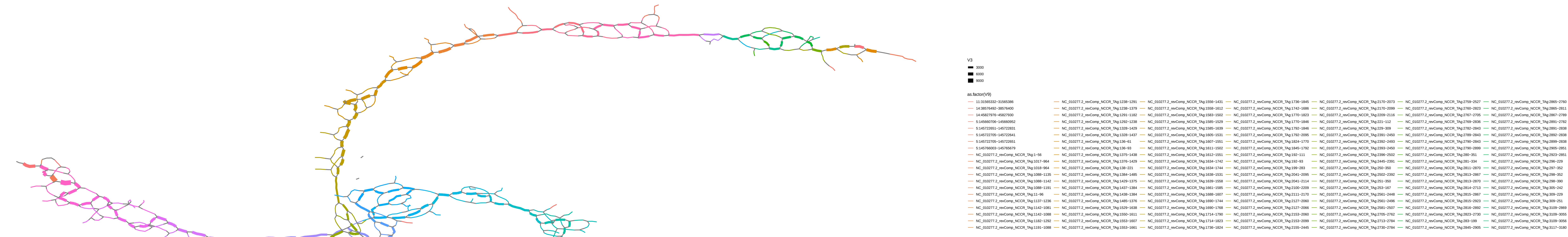
V3
3000
6000
9000
as.factor(V9)
11:31565332−31565386
14:38576492−38576400
14:45827976−45827930
5:145660706−145660952
5:145722651−145722831
NC_010277.2_revComp_NCCR_TAg:1238−1291
NC_010277.2_revComp_NCCR_TAg:1238−1379
NC_010277.2_revComp_NCCR_TAg:1291−1182
NC_010277.2_revComp_NCCR_TAg:1292−1238
NC_010277.2_revComp_NCCR_TAg:1328−1429

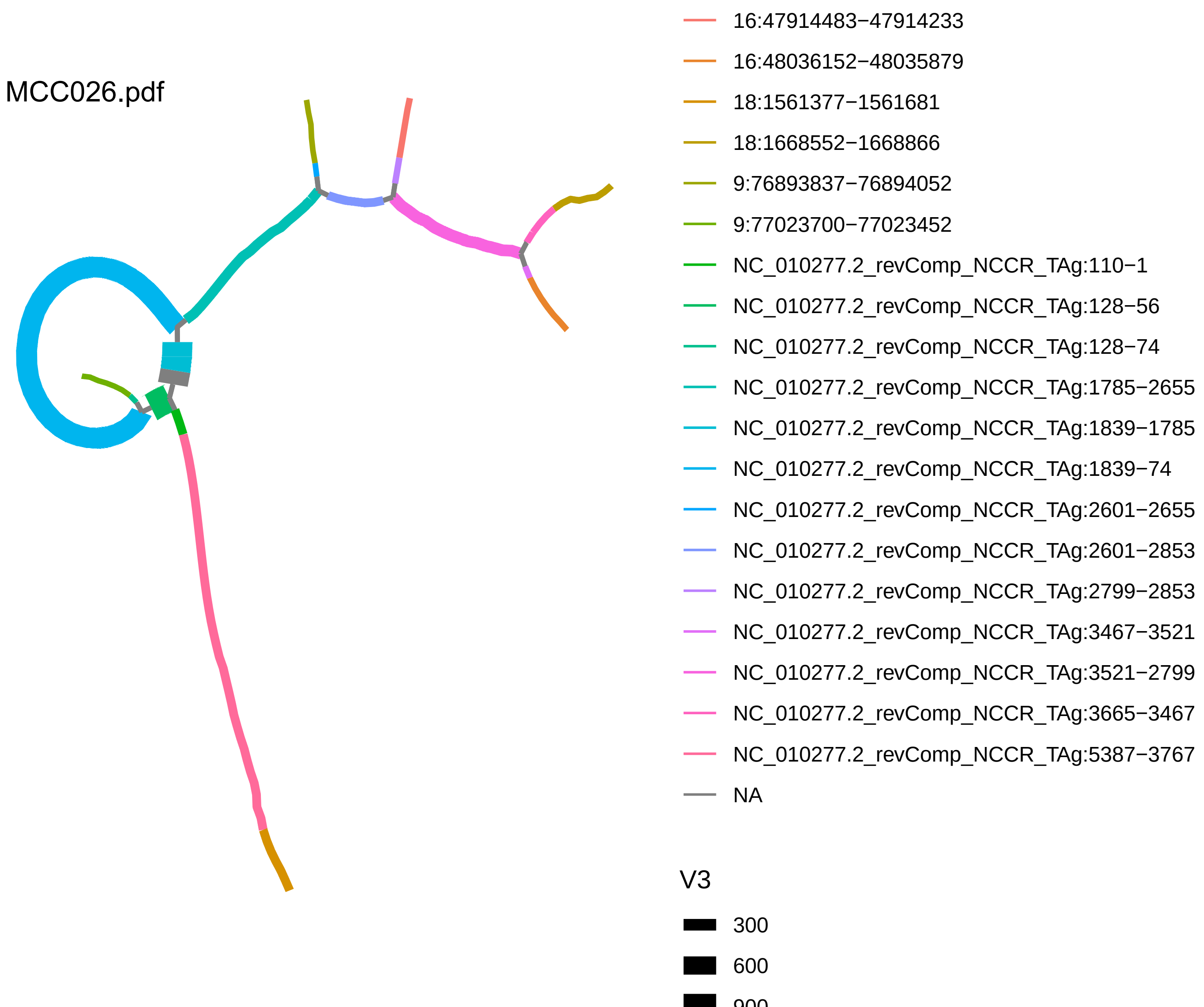
MCC026.pdf
16:47914483−47914233
16:48036152−48035879
18:1561377−1561681
18:1668552−1668866
9:76893837−76894052
9:77023700−77023452
NC_010277.2_revComp_NCCR_TAg:110−1
NC_010277.2_revComp_NCCR_TAg:128−56
NC_010277.2_revComp_NCCR_TAg:128−74
NC_010277.2_revComp_NCCR_TAg:1785−2655
NC_010277.2_revComp_NCCR_TAg:1839−1785
NC_010277.2_revComp_NCCR_TAg:1839−74
NC_010277.2_revComp_NCCR_TAg:2601−2655
NC_010277.2_revComp_NCCR_TAg:2601−2853
NC_010277.2_revComp_NCCR_TAg:2799−2853
NC_010277.2_revComp_NCCR_TAg:3467−3521
NC_010277.2_revComp_NCCR_TAg:3521−2799
NC_010277.2_revComp_NCCR_TAg:3665−3467
NC_010277.2_revComp_NCCR_TAg:5387−3767
NA
V3
300
600

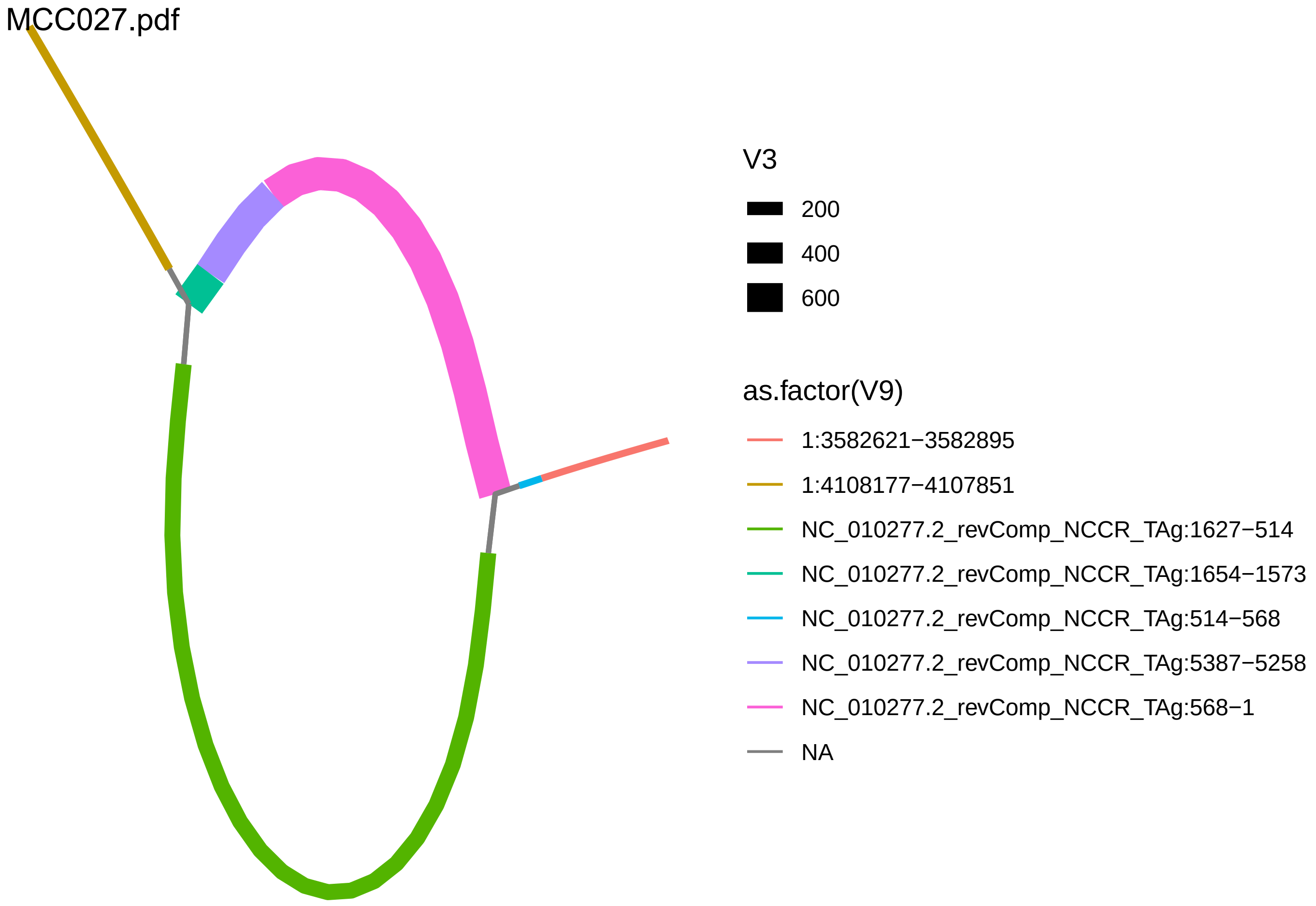
MCC027.pdf
V3
200
400
600
as.factor(V9)
1:3582621−3582895
1:4108177−4107851
NC_010277.2_revComp_NCCR_TAg:1627−514
NC_010277.2_revComp_NCCR_TAg:1654−1573
NC_010277.2_revComp_NCCR_TAg:514−568
NC_010277.2_revComp_NCCR_TAg:5387−5258
NC_010277.2_revComp_NCCR_TAg:568−1
NA

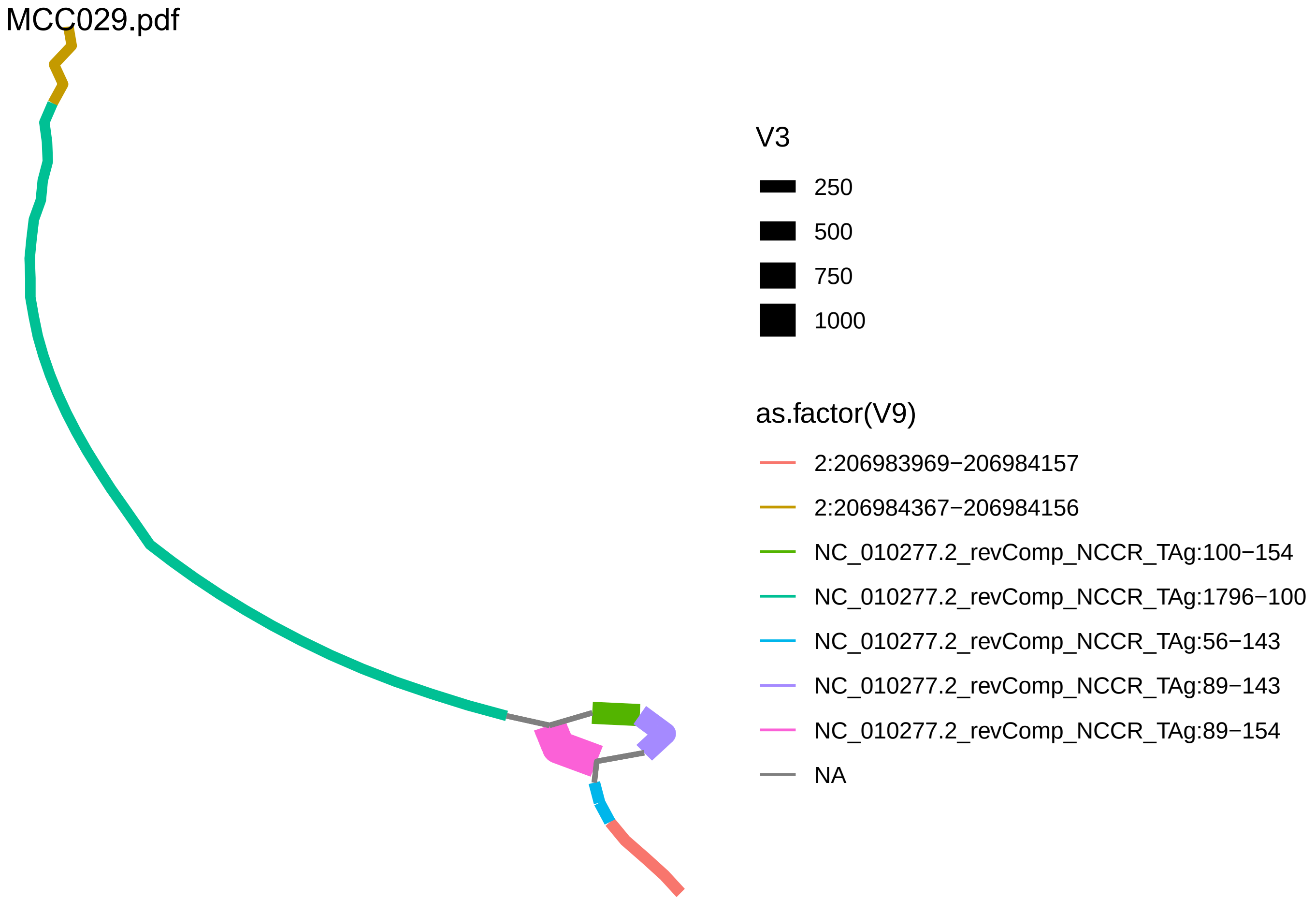
MCC029.pdf
V3
250
500
750
1000
as.factor(V9)
2:206983969−206984157
2:206984367−206984156
NC_010277.2_revComp_NCCR_TAg:100−154
NC_010277.2_revComp_NCCR_TAg:1796−100
NC_010277.2_revComp_NCCR_TAg:56−143
NC_010277.2_revComp_NCCR_TAg:89−143
NC_010277.2_revComp_NCCR_TAg:89−154
NA

MCC036.pdf

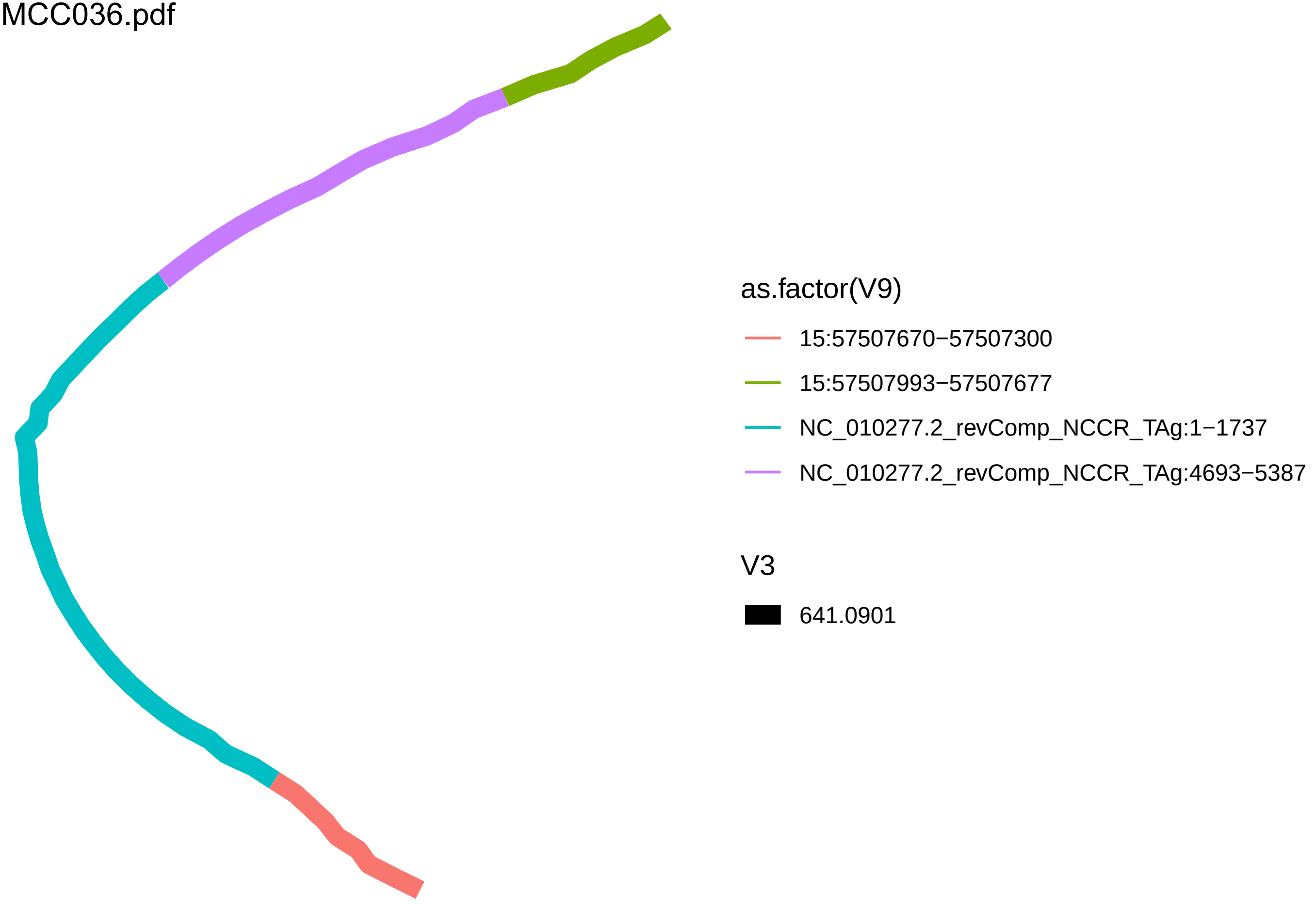
as.factor(V9)
15:57507670−57507300
15:57507993−57507677
NC_010277.2_revComp_NCCR_TAg:1−1737
NC_010277.2_revComp_NCCR_TAg:4693−5387
V3
641.0901

MCC037.pdf

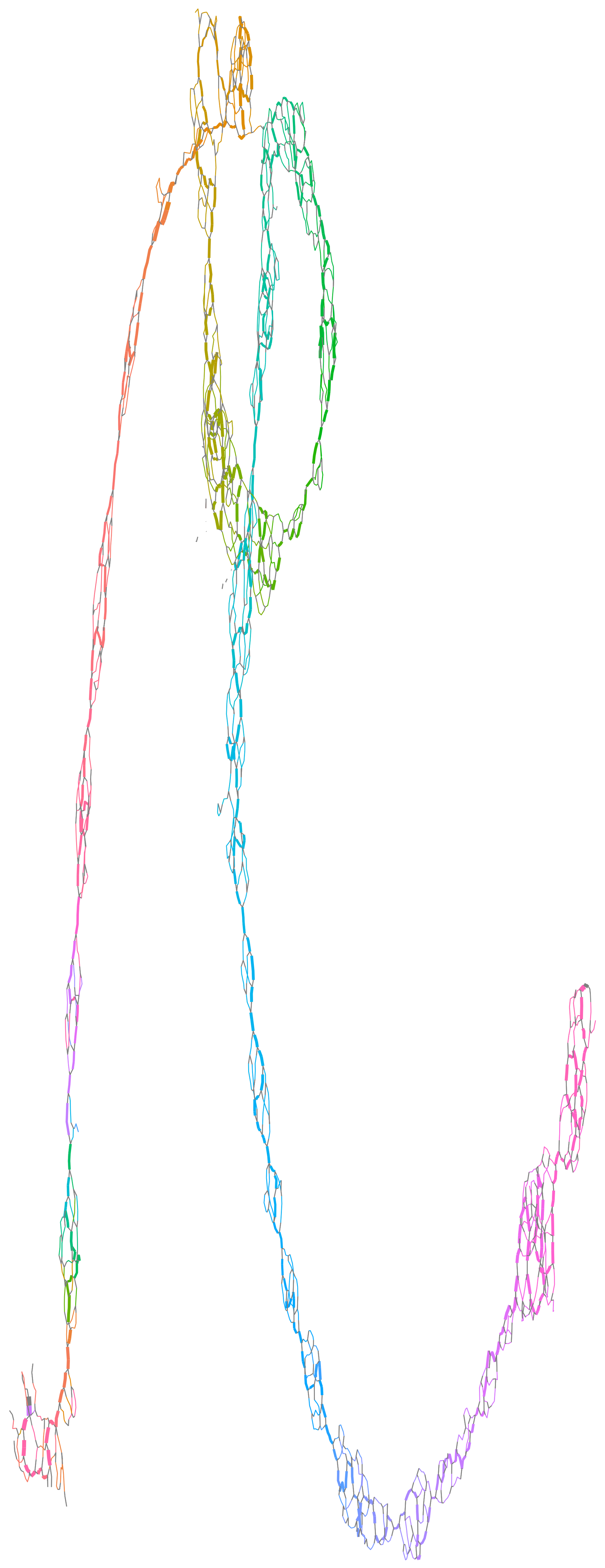

as.factor(V9)
8:20017−20130
NC_010277.2_revComp_NCCR_TAg:1−85
NC_010277.2_revComp_NCCR_TAg:10−113
NC_010277.2_revComp_NCCR_TAg:103−1
NC_010277.2_revComp_NCCR_TAg:1046−1102
NC_010277.2_revComp_NCCR_TAg:1048−1139
NC_010277.2_revComp_NCCR_TAg:1096−1199
NC_010277.2_revComp_NCCR_TAg:1100−1046
NC_010277.2_revComp_NCCR_TAg:1104−1158
NC_010277.2_revComp_NCCR_TAg:111−11
NC_010277.2_revComp_NCCR_TAg:111−9
NC_010277.2_revComp_NCCR_TAg:1141−1195
NC_010277.2_revComp_NCCR_TAg:1150−1085
NC_010277.2_revComp_NCCR_TAg:1156−1048
NC_010277.2_revComp_NCCR_TAg:1156−1096
NC_010277.2_revComp_NCCR_TAg:1158−1102
NC_010277.2_revComp_NCCR_TAg:117−172
NC_010277.2_revComp_NCCR_TAg:118−173
NC_010277.2_revComp_NCCR_TAg:119−197
NC_010277.2_revComp_NCCR_TAg:1193−1085
NC_010277.2_revComp_NCCR_TAg:1195−1104
NC_010277.2_revComp_NCCR_TAg:1199−1141
NC_010277.2_revComp_NCCR_TAg:1206−1141
NC_010277.2_revComp_NCCR_TAg:1206−1145
NC_010277.2_revComp_NCCR_TAg:128−19
NC_010277.2_revComp_NCCR_TAg:129−75
NC_010277.2_revComp_NCCR_TAg:13−122
NC_010277.2_revComp_NCCR_TAg:1306−1152
NC_010277.2_revComp_NCCR_TAg:1306−1252
NC_010277.2_revComp_NCCR_TAg:1309−1252
NC_010277.2_revComp_NCCR_TAg:1309−1255
NC_010277.2_revComp_NCCR_TAg:1310−1255
NC_010277.2_revComp_NCCR_TAg:1310−1256
NC_010277.2_revComp_NCCR_TAg:139−31
NC_010277.2_revComp_NCCR_TAg:139−38
NC_010277.2_revComp_NCCR_TAg:140−38
NC_010277.2_revComp_NCCR_TAg:140−39
NC_010277.2_revComp_NCCR_TAg:141−87
NC_010277.2_revComp_NCCR_TAg:143−197
NC_010277.2_revComp_NCCR_TAg:143−200
NC_010277.2_revComp_NCCR_TAg:146−253
NC_010277.2_revComp_NCCR_TAg:1493−1548
NC_010277.2_revComp_NCCR_TAg:1494−1551
NC_010277.2_revComp_NCCR_TAg:1497−1583
NC_010277.2_revComp_NCCR_TAg:1529−1585
NC_010277.2_revComp_NCCR_TAg:1531−1585
NC_010277.2_revComp_NCCR_TAg:1547−1256
NC_010277.2_revComp_NCCR_TAg:1547−1493
NC_010277.2_revComp_NCCR_TAg:1548−1494
NC_010277.2_revComp_NCCR_TAg:1551−1494
NC_010277.2_revComp_NCCR_TAg:1585−1639
NC_010277.2_revComp_NCCR_TAg:1638−1529
NC_010277.2_revComp_NCCR_TAg:1638−1532
NC_010277.2_revComp_NCCR_TAg:1639−1531
NC_010277.2_revComp_NCCR_TAg:1641−1532
NC_010277.2_revComp_NCCR_TAg:1641−1585
NC_010277.2_revComp_NCCR_TAg:1684−1745
NC_010277.2_revComp_NCCR_TAg:1690−1792
NC_010277.2_revComp_NCCR_TAg:1703−1757
NC_010277.2_revComp_NCCR_TAg:171−66
NC_010277.2_revComp_NCCR_TAg:172−63
NC_010277.2_revComp_NCCR_TAg:173−119
NC_010277.2_revComp_NCCR_TAg:1738−1587
NC_010277.2_revComp_NCCR_TAg:1743−1852
NC_010277.2_revComp_NCCR_TAg:1743−1856
NC_010277.2_revComp_NCCR_TAg:1745−1684
NC_010277.2_revComp_NCCR_TAg:1747−1856
NC_010277.2_revComp_NCCR_TAg:1749−1858
NC_010277.2_revComp_NCCR_TAg:1756−1857
NC_010277.2_revComp_NCCR_TAg:1757−1691
NC_010277.2_revComp_NCCR_TAg:1757−1704
NC_010277.2_revComp_NCCR_TAg:1791−1690
NC_010277.2_revComp_NCCR_TAg:1791−1691
NC_010277.2_revComp_NCCR_TAg:1792−1703
NC_010277.2_revComp_NCCR_TAg:1794−1737
NC_010277.2_revComp_NCCR_TAg:1794−1738
NC_010277.2_revComp_NCCR_TAg:1796−1740
NC_010277.2_revComp_NCCR_TAg:1796−1742
NC_010277.2_revComp_NCCR_TAg:1801−1742
NC_010277.2_revComp_NCCR_TAg:1802−1747
NC_010277.2_revComp_NCCR_TAg:1802−1748
NC_010277.2_revComp_NCCR_TAg:1803−1748
NC_010277.2_revComp_NCCR_TAg:1803−1857
NC_010277.2_revComp_NCCR_TAg:1805−1858
NC_010277.2_revComp_NCCR_TAg:1805−1859
NC_010277.2_revComp_NCCR_TAg:1810−1749
NC_010277.2_revComp_NCCR_TAg:1811−1865
NC_010277.2_revComp_NCCR_TAg:1812−1921
NC_010277.2_revComp_NCCR_TAg:1813−1704
NC_010277.2_revComp_NCCR_TAg:1813−1756
NC_010277.2_revComp_NCCR_TAg:1852−1759
NC_010277.2_revComp_NCCR_TAg:1857−1755
NC_010277.2_revComp_NCCR_TAg:1857−1798
NC_010277.2_revComp_NCCR_TAg:1858−1749
NC_010277.2_revComp_NCCR_TAg:1858−1755
NC_010277.2_revComp_NCCR_TAg:1858−1803
NC_010277.2_revComp_NCCR_TAg:1859−1804
NC_010277.2_revComp_NCCR_TAg:1865−1805
NC_010277.2_revComp_NCCR_TAg:1865−1812
NC_010277.2_revComp_NCCR_TAg:1910−1964
NC_010277.2_revComp_NCCR_TAg:1921−1811
NC_010277.2_revComp_NCCR_TAg:1964−1867
NC_010277.2_revComp_NCCR_TAg:1966−2033
NC_010277.2_revComp_NCCR_TAg:1976−1910
NC_010277.2_revComp_NCCR_TAg:1976−1922
NC_010277.2_revComp_NCCR_TAg:1977−1922
NC_010277.2_revComp_NCCR_TAg:1979−2131
NC_010277.2_revComp_NCCR_TAg:198−299
NC_010277.2_revComp_NCCR_TAg:199−253
NC_010277.2_revComp_NCCR_TAg:2−64
NC_010277.2_revComp_NCCR_TAg:2020−1912
NC_010277.2_revComp_NCCR_TAg:2020−1923
NC_010277.2_revComp_NCCR_TAg:2032−1923
NC_010277.2_revComp_NCCR_TAg:2032−1979
NC_010277.2_revComp_NCCR_TAg:2033−1979
NC_010277.2_revComp_NCCR_TAg:2077−2131
NC_010277.2_revComp_NCCR_TAg:2077−2133
NC_010277.2_revComp_NCCR_TAg:2079−2171
NC_010277.2_revComp_NCCR_TAg:2079−2187
NC_010277.2_revComp_NCCR_TAg:2117−2187
NC_010277.2_revComp_NCCR_TAg:2133−2189
NC_010277.2_revComp_NCCR_TAg:2135−2189
NC_010277.2_revComp_NCCR_TAg:2147−2250
NC_010277.2_revComp_NCCR_TAg:2173−2227
NC_010277.2_revComp_NCCR_TAg:2187−2079
NC_010277.2_revComp_NCCR_TAg:2195−2135
NC_010277.2_revComp_NCCR_TAg:2200−2141
NC_010277.2_revComp_NCCR_TAg:2200−2146
NC_010277.2_revComp_NCCR_TAg:2202−2146
NC_010277.2_revComp_NCCR_TAg:2225−2334
NC_010277.2_revComp_NCCR_TAg:2226−2117
NC_010277.2_revComp_NCCR_TAg:2226−2173
NC_010277.2_revComp_NCCR_TAg:2227−2148
NC_010277.2_revComp_NCCR_TAg:2250−2141
NC_010277.2_revComp_NCCR_TAg:2251−2173
NC_010277.2_revComp_NCCR_TAg:2251−2197
NC_010277.2_revComp_NCCR_TAg:2254−2147
NC_010277.2_revComp_NCCR_TAg:2254−2148
NC_010277.2_revComp_NCCR_TAg:2256−2197
NC_010277.2_revComp_NCCR_TAg:2256−2202
NC_010277.2_revComp_NCCR_TAg:2261−2371
NC_010277.2_revComp_NCCR_TAg:2278−2202
NC_010277.2_revComp_NCCR_TAg:2278−2224
NC_010277.2_revComp_NCCR_TAg:2279−2224
NC_010277.2_revComp_NCCR_TAg:2281−2335
NC_010277.2_revComp_NCCR_TAg:2304−2362
NC_010277.2_revComp_NCCR_TAg:2304−2413
NC_010277.2_revComp_NCCR_TAg:2308−2366
NC_010277.2_revComp_NCCR_TAg:2308−2417
NC_010277.2_revComp_NCCR_TAg:2312−2370
NC_010277.2_revComp_NCCR_TAg:2312−2371
NC_010277.2_revComp_NCCR_TAg:2314−2225
NC_010277.2_revComp_NCCR_TAg:2314−2260
NC_010277.2_revComp_NCCR_TAg:2316−2381
NC_010277.2_revComp_NCCR_TAg:2327−2391
NC_010277.2_revComp_NCCR_TAg:2334−2225
NC_010277.2_revComp_NCCR_TAg:2334−2260
NC_010277.2_revComp_NCCR_TAg:2335−2280
NC_010277.2_revComp_NCCR_TAg:2337−2391
NC_010277.2_revComp_NCCR_TAg:2339−2447
NC_010277.2_revComp_NCCR_TAg:2344−2281
NC_010277.2_revComp_NCCR_TAg:2344−2453
NC_010277.2_revComp_NCCR_TAg:2346−2412
NC_010277.2_revComp_NCCR_TAg:2358−2290
NC_010277.2_revComp_NCCR_TAg:2358−2412
NC_010277.2_revComp_NCCR_TAg:2358−2413
NC_010277.2_revComp_NCCR_TAg:2359−2412
NC_010277.2_revComp_NCCR_TAg:2359−2414
NC_010277.2_revComp_NCCR_TAg:2359−2468
NC_010277.2_revComp_NCCR_TAg:2360−2413
NC_010277.2_revComp_NCCR_TAg:2360−2414
NC_010277.2_revComp_NCCR_TAg:2360−2418
NC_010277.2_revComp_NCCR_TAg:2364−2417
NC_010277.2_revComp_NCCR_TAg:2364−2418
NC_010277.2_revComp_NCCR_TAg:2364−2422
NC_010277.2_revComp_NCCR_TAg:2368−2437
NC_010277.2_revComp_NCCR_TAg:2370−2261
NC_010277.2_revComp_NCCR_TAg:2383−2437
NC_010277.2_revComp_NCCR_TAg:2383−2441
NC_010277.2_revComp_NCCR_TAg:2387−2445
NC_010277.2_revComp_NCCR_TAg:2388−2496
NC_010277.2_revComp_NCCR_TAg:2391−2446
NC_010277.2_revComp_NCCR_TAg:2392−2447
NC_010277.2_revComp_NCCR_TAg:2392−2501
NC_010277.2_revComp_NCCR_TAg:2393−2337
NC_010277.2_revComp_NCCR_TAg:2393−2449
NC_010277.2_revComp_NCCR_TAg:2395−2453
NC_010277.2_revComp_NCCR_TAg:2396−2501
NC_010277.2_revComp_NCCR_TAg:2397−2339
NC_010277.2_revComp_NCCR_TAg:2397−2343
NC_010277.2_revComp_NCCR_TAg:2398−2290
NC_010277.2_revComp_NCCR_TAg:2398−2343
NC_010277.2_revComp_NCCR_TAg:2398−2346
NC_010277.2_revComp_NCCR_TAg:2399−2454
NC_010277.2_revComp_NCCR_TAg:2400−2344
NC_010277.2_revComp_NCCR_TAg:2400−2346
NC_010277.2_revComp_NCCR_TAg:2400−2468
NC_010277.2_revComp_NCCR_TAg:2413−2304
NC_010277.2_revComp_NCCR_TAg:2413−2359
NC_010277.2_revComp_NCCR_TAg:2413−2360
NC_010277.2_revComp_NCCR_TAg:2414−2469
NC_010277.2_revComp_NCCR_TAg:2422−2317
NC_010277.2_revComp_NCCR_TAg:2435−2327
NC_010277.2_revComp_NCCR_TAg:2435−2364
NC_010277.2_revComp_NCCR_TAg:2437−2491
NC_010277.2_revComp_NCCR_TAg:2439−2547
NC_010277.2_revComp_NCCR_TAg:2441−2387
NC_010277.2_revComp_NCCR_TAg:2443−2497
NC_010277.2_revComp_NCCR_TAg:2445−2391
NC_010277.2_revComp_NCCR_TAg:2447−2339
NC_010277.2_revComp_NCCR_TAg:2449−2395
NC_010277.2_revComp_NCCR_TAg:2451−2392
NC_010277.2_revComp_NCCR_TAg:2453−2344
NC_010277.2_revComp_NCCR_TAg:2454−2400
NC_010277.2_revComp_NCCR_TAg:2458−2512
NC_010277.2_revComp_NCCR_TAg:2459−2512
NC_010277.2_revComp_NCCR_TAg:2459−2568
NC_010277.2_revComp_NCCR_TAg:2469−2360
NC_010277.2_revComp_NCCR_TAg:2479−2533
NC_010277.2_revComp_NCCR_TAg:2480−2589
NC_010277.2_revComp_NCCR_TAg:2486−2549
NC_010277.2_revComp_NCCR_TAg:2491−2415
NC_010277.2_revComp_NCCR_TAg:2491−2439
NC_010277.2_revComp_NCCR_TAg:2495−2596
NC_010277.2_revComp_NCCR_TAg:2496−2387
NC_010277.2_revComp_NCCR_TAg:2497−2388
NC_010277.2_revComp_NCCR_TAg:2497−2437
NC_010277.2_revComp_NCCR_TAg:2501−2392
NC_010277.2_revComp_NCCR_TAg:2501−2396
NC_010277.2_revComp_NCCR_TAg:2501−2443
NC_010277.2_revComp_NCCR_TAg:2501−2555
NC_010277.2_revComp_NCCR_TAg:2502−2447
NC_010277.2_revComp_NCCR_TAg:2502−2448
NC_010277.2_revComp_NCCR_TAg:251−350
NC_010277.2_revComp_NCCR_TAg:2512−2448
NC_010277.2_revComp_NCCR_TAg:2514−2589
NC_010277.2_revComp_NCCR_TAg:252−146
NC_010277.2_revComp_NCCR_TAg:253−146
NC_010277.2_revComp_NCCR_TAg:253−198
NC_010277.2_revComp_NCCR_TAg:253−199
NC_010277.2_revComp_NCCR_TAg:2533−2458
NC_010277.2_revComp_NCCR_TAg:2534−2479
NC_010277.2_revComp_NCCR_TAg:2535−2590
NC_010277.2_revComp_NCCR_TAg:2536−2590
NC_010277.2_revComp_NCCR_TAg:2536−2596
NC_010277.2_revComp_NCCR_TAg:2540−2480
NC_010277.2_revComp_NCCR_TAg:2542−2605
NC_010277.2_revComp_NCCR_TAg:2547−2486
NC_010277.2_revComp_NCCR_TAg:2549−2493
NC_010277.2_revComp_NCCR_TAg:2551−2611
NC_010277.2_revComp_NCCR_TAg:2555−2495
NC_010277.2_revComp_NCCR_TAg:2557−2612
NC_010277.2_revComp_NCCR_TAg:2558−2612
NC_010277.2_revComp_NCCR_TAg:2558−2614
NC_010277.2_revComp_NCCR_TAg:256−199
NC_010277.2_revComp_NCCR_TAg:2560−2668
NC_010277.2_revComp_NCCR_TAg:2568−2501
NC_010277.2_revComp_NCCR_TAg:2589−2480
NC_010277.2_revComp_NCCR_TAg:2596−2495
NC_010277.2_revComp_NCCR_TAg:2605−2542
NC_010277.2_revComp_NCCR_TAg:2611−2502
NC_010277.2_revComp_NCCR_TAg:2614−2670
NC_010277.2_revComp_NCCR_TAg:2616−2670
NC_010277.2_revComp_NCCR_TAg:2616−2726
NC_010277.2_revComp_NCCR_TAg:2668−2560
NC_010277.2_revComp_NCCR_TAg:2672−2781
NC_010277.2_revComp_NCCR_TAg:2672−2782
NC_010277.2_revComp_NCCR_TAg:2726−2672
NC_010277.2_revComp_NCCR_TAg:2728−2782
NC_010277.2_revComp_NCCR_TAg:2737−2791
NC_010277.2_revComp_NCCR_TAg:2738−2847
NC_010277.2_revComp_NCCR_TAg:2747−2801
NC_010277.2_revComp_NCCR_TAg:2781−2673
NC_010277.2_revComp_NCCR_TAg:2782−2673
NC_010277.2_revComp_NCCR_TAg:2784−2893
NC_010277.2_revComp_NCCR_TAg:2791−2728
NC_010277.2_revComp_NCCR_TAg:2792−2737
NC_010277.2_revComp_NCCR_TAg:2801−2738
NC_010277.2_revComp_NCCR_TAg:2803−2747
NC_010277.2_revComp_NCCR_TAg:2804−2749
NC_010277.2_revComp_NCCR_TAg:2805−2858
NC_010277.2_revComp_NCCR_TAg:2837−2750
NC_010277.2_revComp_NCCR_TAg:2837−2783
NC_010277.2_revComp_NCCR_TAg:2838−2783
NC_010277.2_revComp_NCCR_TAg:2838−2947
NC_010277.2_revComp_NCCR_TAg:2840−2894
NC_010277.2_revComp_NCCR_TAg:2847−2738
NC_010277.2_revComp_NCCR_TAg:2847−2784
NC_010277.2_revComp_NCCR_TAg:2848−2793
NC_010277.2_revComp_NCCR_TAg:2848−2794
NC_010277.2_revComp_NCCR_TAg:2857−2749
NC_010277.2_revComp_NCCR_TAg:2857−2794
NC_010277.2_revComp_NCCR_TAg:2858−2750
NC_010277.2_revComp_NCCR_TAg:2859−2803
NC_010277.2_revComp_NCCR_TAg:2859−2805
NC_010277.2_revComp_NCCR_TAg:2892−2805
NC_010277.2_revComp_NCCR_TAg:2893−2784
NC_010277.2_revComp_NCCR_TAg:2893−2838
NC_010277.2_revComp_NCCR_TAg:2894−2839
NC_010277.2_revComp_NCCR_TAg:2894−2947
NC_010277.2_revComp_NCCR_TAg:2894−2948
NC_010277.2_revComp_NCCR_TAg:2906−2960
NC_010277.2_revComp_NCCR_TAg:2907−2960
NC_010277.2_revComp_NCCR_TAg:2907−3016
NC_010277.2_revComp_NCCR_TAg:2908−3010
NC_010277.2_revComp_NCCR_TAg:2908−3011
NC_010277.2_revComp_NCCR_TAg:2913−3022
NC_010277.2_revComp_NCCR_TAg:2915−3023
NC_010277.2_revComp_NCCR_TAg:2947−2840
NC_010277.2_revComp_NCCR_TAg:2947−2893
NC_010277.2_revComp_NCCR_TAg:2947−2895
NC_010277.2_revComp_NCCR_TAg:2948−2893
NC_010277.2_revComp_NCCR_TAg:296−202
NC_010277.2_revComp_NCCR_TAg:2960−2894
NC_010277.2_revComp_NCCR_TAg:2960−2906
NC_010277.2_revComp_NCCR_TAg:2961−2906
NC_010277.2_revComp_NCCR_TAg:2966−3020
NC_010277.2_revComp_NCCR_TAg:2967−2907
NC_010277.2_revComp_NCCR_TAg:2967−2913
NC_010277.2_revComp_NCCR_TAg:2967−3076
NC_010277.2_revComp_NCCR_TAg:2969−3023
NC_010277.2_revComp_NCCR_TAg:298−200
NC_010277.2_revComp_NCCR_TAg:298−242
NC_010277.2_revComp_NCCR_TAg:298−352
NC_010277.2_revComp_NCCR_TAg:298−421
NC_010277.2_revComp_NCCR_TAg:299−200
NC_010277.2_revComp_NCCR_TAg:299−201
NC_010277.2_revComp_NCCR_TAg:299−244
NC_010277.2_revComp_NCCR_TAg:2997−2913
NC_010277.2_revComp_NCCR_TAg:2997−2943
NC_010277.2_revComp_NCCR_TAg:2998−2943
NC_010277.2_revComp_NCCR_TAg:3000−3054
NC_010277.2_revComp_NCCR_TAg:3003−2895
NC_010277.2_revComp_NCCR_TAg:3003−2944
NC_010277.2_revComp_NCCR_TAg:3006−3115
NC_010277.2_revComp_NCCR_TAg:3010−2949
NC_010277.2_revComp_NCCR_TAg:3011−2907
NC_010277.2_revComp_NCCR_TAg:3016−2956
NC_010277.2_revComp_NCCR_TAg:3020−2962
NC_010277.2_revComp_NCCR_TAg:3021−2966
NC_010277.2_revComp_NCCR_TAg:3022−2915
NC_010277.2_revComp_NCCR_TAg:3023−2967
NC_010277.2_revComp_NCCR_TAg:3023−3077
NC_010277.2_revComp_NCCR_TAg:3038−3147
NC_010277.2_revComp_NCCR_TAg:305−242
NC_010277.2_revComp_NCCR_TAg:305−245
NC_010277.2_revComp_NCCR_TAg:3050−3116
NC_010277.2_revComp_NCCR_TAg:3052−2944
NC_010277.2_revComp_NCCR_TAg:3052−2948
NC_010277.2_revComp_NCCR_TAg:3052−2969
NC_010277.2_revComp_NCCR_TAg:3053−2944
NC_010277.2_revComp_NCCR_TAg:3053−2998
NC_010277.2_revComp_NCCR_TAg:3054−2999
NC_010277.2_revComp_NCCR_TAg:3060−3000
NC_010277.2_revComp_NCCR_TAg:3062−3115
NC_010277.2_revComp_NCCR_TAg:3062−3116
NC_010277.2_revComp_NCCR_TAg:3062−3122
NC_010277.2_revComp_NCCR_TAg:3064−3006
NC_010277.2_revComp_NCCR_TAg:3068−3122
NC_010277.2_revComp_NCCR_TAg:3068−3148
NC_010277.2_revComp_NCCR_TAg:3076−2967
NC_010277.2_revComp_NCCR_TAg:3076−3006
NC_010277.2_revComp_NCCR_TAg:3077−3022
NC_010277.2_revComp_NCCR_TAg:309−201
NC_010277.2_revComp_NCCR_TAg:309−244
NC_010277.2_revComp_NCCR_TAg:309−251
NC_010277.2_revComp_NCCR_TAg:3092−3023
NC_010277.2_revComp_NCCR_TAg:3094−3147
NC_010277.2_revComp_NCCR_TAg:3094−3148
NC_010277.2_revComp_NCCR_TAg:3094−3156
NC_010277.2_revComp_NCCR_TAg:3102−3160
NC_010277.2_revComp_NCCR_TAg:3103−3156
NC_010277.2_revComp_NCCR_TAg:3103−3212
NC_010277.2_revComp_NCCR_TAg:3104−3038
NC_010277.2_revComp_NCCR_TAg:3106−3173
NC_010277.2_revComp_NCCR_TAg:3112−3050
NC_010277.2_revComp_NCCR_TAg:3116−3010
NC_010277.2_revComp_NCCR_TAg:3119−3173
NC_010277.2_revComp_NCCR_TAg:3119−3199
NC_010277.2_revComp_NCCR_TAg:3121−3058
NC_010277.2_revComp_NCCR_TAg:3122−3077
NC_010277.2_revComp_NCCR_TAg:3131−3094
NC_010277.2_revComp_NCCR_TAg:3145−3201
NC_010277.2_revComp_NCCR_TAg:3145−3253
NC_010277.2_revComp_NCCR_TAg:3147−3204
NC_010277.2_revComp_NCCR_TAg:3150−3212
NC_010277.2_revComp_NCCR_TAg:3156−3102
NC_010277.2_revComp_NCCR_TAg:3158−3229
NC_010277.2_revComp_NCCR_TAg:3158−3265
NC_010277.2_revComp_NCCR_TAg:3160−3058
NC_010277.2_revComp_NCCR_TAg:3175−3233
NC_010277.2_revComp_NCCR_TAg:3179−3235
NC_010277.2_revComp_NCCR_TAg:3180−3233
NC_010277.2_revComp_NCCR_TAg:3181−3255
NC_010277.2_revComp_NCCR_TAg:3182−3291
NC_010277.2_revComp_NCCR_TAg:3201−3253
NC_010277.2_revComp_NCCR_TAg:3201−3257
NC_010277.2_revComp_NCCR_TAg:3203−3268
NC_010277.2_revComp_NCCR_TAg:3204−3150
NC_010277.2_revComp_NCCR_TAg:3214−3274
NC_010277.2_revComp_NCCR_TAg:3221−3330
NC_010277.2_revComp_NCCR_TAg:3228−3119
NC_010277.2_revComp_NCCR_TAg:3228−3120
NC_010277.2_revComp_NCCR_TAg:3229−3120
NC_010277.2_revComp_NCCR_TAg:3229−3175
NC_010277.2_revComp_NCCR_TAg:3233−3179
NC_010277.2_revComp_NCCR_TAg:3235−3147
NC_010277.2_revComp_NCCR_TAg:3235−3152
NC_010277.2_revComp_NCCR_TAg:3235−3181
NC_010277.2_revComp_NCCR_TAg:3237−3330
NC_010277.2_revComp_NCCR_TAg:3255−3201
NC_010277.2_revComp_NCCR_TAg:3257−3152
NC_010277.2_revComp_NCCR_TAg:3268−3211
NC_010277.2_revComp_NCCR_TAg:3274−3220
NC_010277.2_revComp_NCCR_TAg:3276−3330
NC_010277.2_revComp_NCCR_TAg:3276−3442
NC_010277.2_revComp_NCCR_TAg:3286−3180
NC_010277.2_revComp_NCCR_TAg:3286−3220
NC_010277.2_revComp_NCCR_TAg:3291−3232
NC_010277.2_revComp_NCCR_TAg:3329−3220
NC_010277.2_revComp_NCCR_TAg:3329−3221
NC_010277.2_revComp_NCCR_TAg:3388−3442
NC_010277.2_revComp_NCCR_TAg:3389−3498
NC_010277.2_revComp_NCCR_TAg:3403−3511
NC_010277.2_revComp_NCCR_TAg:3412−3466
NC_010277.2_revComp_NCCR_TAg:3443−3388
NC_010277.2_revComp_NCCR_TAg:3444−3499
NC_010277.2_revComp_NCCR_TAg:3445−3499
NC_010277.2_revComp_NCCR_TAg:3445−3510
NC_010277.2_revComp_NCCR_TAg:3454−3389
NC_010277.2_revComp_NCCR_TAg:3454−3400
NC_010277.2_revComp_NCCR_TAg:3456−3400
NC_010277.2_revComp_NCCR_TAg:3456−3511
NC_010277.2_revComp_NCCR_TAg:3457−3511
NC_010277.2_revComp_NCCR_TAg:3457−3512
NC_010277.2_revComp_NCCR_TAg:3458−3512
NC_010277.2_revComp_NCCR_TAg:3458−3522
NC_010277.2_revComp_NCCR_TAg:3458−3564
NC_010277.2_revComp_NCCR_TAg:3466−3402
NC_010277.2_revComp_NCCR_TAg:3468−3412
NC_010277.2_revComp_NCCR_TAg:3468−3524
NC_010277.2_revComp_NCCR_TAg:3470−3524
NC_010277.2_revComp_NCCR_TAg:3470−3567
NC_010277.2_revComp_NCCR_TAg:3498−3389
NC_010277.2_revComp_NCCR_TAg:3498−3414
NC_010277.2_revComp_NCCR_TAg:350−250
NC_010277.2_revComp_NCCR_TAg:351−250
NC_010277.2_revComp_NCCR_TAg:351−255
NC_010277.2_revComp_NCCR_TAg:3510−3403
NC_010277.2_revComp_NCCR_TAg:3511−3402
NC_010277.2_revComp_NCCR_TAg:3514−3568
NC_010277.2_revComp_NCCR_TAg:3514−3623
NC_010277.2_revComp_NCCR_TAg:352−297
NC_010277.2_revComp_NCCR_TAg:3522−3413
NC_010277.2_revComp_NCCR_TAg:3523−3413
NC_010277.2_revComp_NCCR_TAg:3523−3414
NC_010277.2_revComp_NCCR_TAg:3530−3633
NC_010277.2_revComp_NCCR_TAg:3544−3593
NC_010277.2_revComp_NCCR_TAg:3562−3615
NC_010277.2_revComp_NCCR_TAg:3564−3458
NC_010277.2_revComp_NCCR_TAg:3567−3458
NC_010277.2_revComp_NCCR_TAg:3567−3509
NC_010277.2_revComp_NCCR_TAg:3567−3513
NC_010277.2_revComp_NCCR_TAg:3568−3513
NC_010277.2_revComp_NCCR_TAg:3577−3514
NC_010277.2_revComp_NCCR_TAg:3577−3523
NC_010277.2_revComp_NCCR_TAg:3579−3634
NC_010277.2_revComp_NCCR_TAg:3580−3643
NC_010277.2_revComp_NCCR_TAg:3584−3523
NC_010277.2_revComp_NCCR_TAg:3589−3661
NC_010277.2_revComp_NCCR_TAg:3589−3698
NC_010277.2_revComp_NCCR_TAg:3607−3661
NC_010277.2_revComp_NCCR_TAg:3607−3662
NC_010277.2_revComp_NCCR_TAg:3608−3670
NC_010277.2_revComp_NCCR_TAg:3608−3716
NC_010277.2_revComp_NCCR_TAg:3609−3716
NC_010277.2_revComp_NCCR_TAg:3615−3530
NC_010277.2_revComp_NCCR_TAg:3615−3561
NC_010277.2_revComp_NCCR_TAg:3615−3567
NC_010277.2_revComp_NCCR_TAg:3616−3699
NC_010277.2_revComp_NCCR_TAg:3623−3514
NC_010277.2_revComp_NCCR_TAg:3623−3561
NC_010277.2_revComp_NCCR_TAg:3624−3569
NC_010277.2_revComp_NCCR_TAg:3624−3570
NC_010277.2_revComp_NCCR_TAg:3633−3524
NC_010277.2_revComp_NCCR_TAg:3633−3570
NC_010277.2_revComp_NCCR_TAg:3634−3580
NC_010277.2_revComp_NCCR_TAg:3643−3589
NC_010277.2_revComp_NCCR_TAg:3645−3699
NC_010277.2_revComp_NCCR_TAg:3645−3716
NC_010277.2_revComp_NCCR_TAg:3662−3717
NC_010277.2_revComp_NCCR_TAg:3663−3804
NC_010277.2_revComp_NCCR_TAg:367−421
NC_010277.2_revComp_NCCR_TAg:3670−3562
NC_010277.2_revComp_NCCR_TAg:368−477
NC_010277.2_revComp_NCCR_TAg:3698−3590
NC_010277.2_revComp_NCCR_TAg:3699−3590
NC_010277.2_revComp_NCCR_TAg:3717−3663
NC_010277.2_revComp_NCCR_TAg:3750−3804
NC_010277.2_revComp_NCCR_TAg:3751−3855
NC_010277.2_revComp_NCCR_TAg:3752−3855
NC_010277.2_revComp_NCCR_TAg:3800−3860
NC_010277.2_revComp_NCCR_TAg:3805−3750
NC_010277.2_revComp_NCCR_TAg:3806−3892
NC_010277.2_revComp_NCCR_TAg:3838−3893
NC_010277.2_revComp_NCCR_TAg:3839−3918
NC_010277.2_revComp_NCCR_TAg:3839−3948
NC_010277.2_revComp_NCCR_TAg:3854−3751
NC_010277.2_revComp_NCCR_TAg:3854−3752
NC_010277.2_revComp_NCCR_TAg:3864−3918
NC_010277.2_revComp_NCCR_TAg:3864−3919
NC_010277.2_revComp_NCCR_TAg:3865−3948
NC_010277.2_revComp_NCCR_TAg:3865−3974
NC_010277.2_revComp_NCCR_TAg:3892−3838
NC_010277.2_revComp_NCCR_TAg:3921−3990
NC_010277.2_revComp_NCCR_TAg:3936−3994
NC_010277.2_revComp_NCCR_TAg:3940−4015
NC_010277.2_revComp_NCCR_TAg:3943−4050
NC_010277.2_revComp_NCCR_TAg:3948−3839
NC_010277.2_revComp_NCCR_TAg:3949−3894
NC_010277.2_revComp_NCCR_TAg:3949−3895
NC_010277.2_revComp_NCCR_TAg:3961−4015
NC_010277.2_revComp_NCCR_TAg:3961−4046
NC_010277.2_revComp_NCCR_TAg:3974−3895
NC_010277.2_revComp_NCCR_TAg:3975−3920
NC_010277.2_revComp_NCCR_TAg:3975−3921
NC_010277.2_revComp_NCCR_TAg:3990−3936
NC_010277.2_revComp_NCCR_TAg:3992−4046
NC_010277.2_revComp_NCCR_TAg:3994−3940
NC_010277.2_revComp_NCCR_TAg:3994−3943
NC_010277.2_revComp_NCCR_TAg:3996−4076
NC_010277.2_revComp_NCCR_TAg:4023−4076
NC_010277.2_revComp_NCCR_TAg:4023−4126
NC_010277.2_revComp_NCCR_TAg:4023−4132
NC_010277.2_revComp_NCCR_TAg:4024−4133
NC_010277.2_revComp_NCCR_TAg:4026−4132
NC_010277.2_revComp_NCCR_TAg:4026−4133
NC_010277.2_revComp_NCCR_TAg:4045−3936
NC_010277.2_revComp_NCCR_TAg:4045−3937
NC_010277.2_revComp_NCCR_TAg:4046−3937
NC_010277.2_revComp_NCCR_TAg:4050−3992
NC_010277.2_revComp_NCCR_TAg:4072−4133
NC_010277.2_revComp_NCCR_TAg:4076−4022
NC_010277.2_revComp_NCCR_TAg:4077−4022
NC_010277.2_revComp_NCCR_TAg:4078−4023
NC_010277.2_revComp_NCCR_TAg:4079−4133
NC_010277.2_revComp_NCCR_TAg:4079−4134
NC_010277.2_revComp_NCCR_TAg:4080−4134
NC_010277.2_revComp_NCCR_TAg:4080−4137
NC_010277.2_revComp_NCCR_TAg:4083−4137
NC_010277.2_revComp_NCCR_TAg:4083−4138
NC_010277.2_revComp_NCCR_TAg:4084−4156
NC_010277.2_revComp_NCCR_TAg:4084−4188
NC_010277.2_revComp_NCCR_TAg:4103−4212
NC_010277.2_revComp_NCCR_TAg:4126−4024
NC_010277.2_revComp_NCCR_TAg:4140−4212
NC_010277.2_revComp_NCCR_TAg:4156−4102
NC_010277.2_revComp_NCCR_TAg:4157−4102
NC_010277.2_revComp_NCCR_TAg:4158−4213
NC_010277.2_revComp_NCCR_TAg:4159−4311
NC_010277.2_revComp_NCCR_TAg:4188−4084
NC_010277.2_revComp_NCCR_TAg:4193−4084
NC_010277.2_revComp_NCCR_TAg:4193−4103
NC_010277.2_revComp_NCCR_TAg:4193−4133
NC_010277.2_revComp_NCCR_TAg:4194−4139
NC_010277.2_revComp_NCCR_TAg:4194−4140
NC_010277.2_revComp_NCCR_TAg:421−368
NC_010277.2_revComp_NCCR_TAg:4213−4159
NC_010277.2_revComp_NCCR_TAg:4257−4311
NC_010277.2_revComp_NCCR_TAg:4257−4319
NC_010277.2_revComp_NCCR_TAg:4258−4319
NC_010277.2_revComp_NCCR_TAg:4266−4375
NC_010277.2_revComp_NCCR_TAg:4269−4327
NC_010277.2_revComp_NCCR_TAg:4269−4358
NC_010277.2_revComp_NCCR_TAg:4304−4358
NC_010277.2_revComp_NCCR_TAg:4304−4367
NC_010277.2_revComp_NCCR_TAg:4305−4414
NC_010277.2_revComp_NCCR_TAg:4313−4375
NC_010277.2_revComp_NCCR_TAg:4319−4265
NC_010277.2_revComp_NCCR_TAg:4319−4266
NC_010277.2_revComp_NCCR_TAg:4321−4379
NC_010277.2_revComp_NCCR_TAg:4322−4379
NC_010277.2_revComp_NCCR_TAg:4323−4265
NC_010277.2_revComp_NCCR_TAg:4325−4390
NC_010277.2_revComp_NCCR_TAg:4336−4390
NC_010277.2_revComp_NCCR_TAg:4336−4406
NC_010277.2_revComp_NCCR_TAg:4337−4446
NC_010277.2_revComp_NCCR_TAg:4352−4406
NC_010277.2_revComp_NCCR_TAg:4352−4414
NC_010277.2_revComp_NCCR_TAg:4353−4462
NC_010277.2_revComp_NCCR_TAg:4358−4305
NC_010277.2_revComp_NCCR_TAg:4360−4431
NC_010277.2_revComp_NCCR_TAg:4367−4258
NC_010277.2_revComp_NCCR_TAg:437−542
NC_010277.2_revComp_NCCR_TAg:4375−4321
NC_010277.2_revComp_NCCR_TAg:4377−4446
NC_010277.2_revComp_NCCR_TAg:4379−4273
NC_010277.2_revComp_NCCR_TAg:4390−4336
NC_010277.2_revComp_NCCR_TAg:4390−4337
NC_010277.2_revComp_NCCR_TAg:4392−4459
NC_010277.2_revComp_NCCR_TAg:4405−4462
NC_010277.2_revComp_NCCR_TAg:4408−4463
NC_010277.2_revComp_NCCR_TAg:4409−4463
NC_010277.2_revComp_NCCR_TAg:4410−4514
NC_010277.2_revComp_NCCR_TAg:4410−4519
NC_010277.2_revComp_NCCR_TAg:4431−4322
NC_010277.2_revComp_NCCR_TAg:4461−4515
NC_010277.2_revComp_NCCR_TAg:4514−4405
NC_010277.2_revComp_NCCR_TAg:4515−4409
NC_010277.2_revComp_NCCR_TAg:4519−4461
NC_010277.2_revComp_NCCR_TAg:4548−4602
NC_010277.2_revComp_NCCR_TAg:4548−4603
NC_010277.2_revComp_NCCR_TAg:4549−4654
NC_010277.2_revComp_NCCR_TAg:4549−4658
NC_010277.2_revComp_NCCR_TAg:455−509
NC_010277.2_revComp_NCCR_TAg:458−565
NC_010277.2_revComp_NCCR_TAg:4601−4655
NC_010277.2_revComp_NCCR_TAg:4602−4465
NC_010277.2_revComp_NCCR_TAg:4602−4707
NC_010277.2_revComp_NCCR_TAg:4602−4711
NC_010277.2_revComp_NCCR_TAg:4603−4707
NC_010277.2_revComp_NCCR_TAg:4605−4659
NC_010277.2_revComp_NCCR_TAg:4605−4711
NC_010277.2_revComp_NCCR_TAg:4652−4711
NC_010277.2_revComp_NCCR_TAg:4654−4549
NC_010277.2_revComp_NCCR_TAg:4655−4549
NC_010277.2_revComp_NCCR_TAg:4656−4601
NC_010277.2_revComp_NCCR_TAg:4657−4602
NC_010277.2_revComp_NCCR_TAg:4657−4712
NC_010277.2_revComp_NCCR_TAg:4658−4549
NC_010277.2_revComp_NCCR_TAg:4658−4599
NC_010277.2_revComp_NCCR_TAg:4658−4603
NC_010277.2_revComp_NCCR_TAg:4658−4712
NC_010277.2_revComp_NCCR_TAg:4658−4729
NC_010277.2_revComp_NCCR_TAg:4659−4604
NC_010277.2_revComp_NCCR_TAg:4675−4729
NC_010277.2_revComp_NCCR_TAg:4675−4730
NC_010277.2_revComp_NCCR_TAg:4676−4785
NC_010277.2_revComp_NCCR_TAg:471−524
NC_010277.2_revComp_NCCR_TAg:4711−4602
NC_010277.2_revComp_NCCR_TAg:4729−4676
NC_010277.2_revComp_NCCR_TAg:4759−4813
NC_010277.2_revComp_NCCR_TAg:4760−4824
NC_010277.2_revComp_NCCR_TAg:4760−4869
NC_010277.2_revComp_NCCR_TAg:477−367
NC_010277.2_revComp_NCCR_TAg:4784−4676
NC_010277.2_revComp_NCCR_TAg:4785−4731
NC_010277.2_revComp_NCCR_TAg:4802−4869
NC_010277.2_revComp_NCCR_TAg:4802−4911
NC_010277.2_revComp_NCCR_TAg:4813−4731
NC_010277.2_revComp_NCCR_TAg:4815−4872
NC_010277.2_revComp_NCCR_TAg:4818−4912
NC_010277.2_revComp_NCCR_TAg:4856−4759
NC_010277.2_revComp_NCCR_TAg:4858−4912
NC_010277.2_revComp_NCCR_TAg:4858−4928
NC_010277.2_revComp_NCCR_TAg:4874−4926
NC_010277.2_revComp_NCCR_TAg:4874−4928
NC_010277.2_revComp_NCCR_TAg:488−423
NC_010277.2_revComp_NCCR_TAg:488−434
NC_010277.2_revComp_NCCR_TAg:488−544
NC_010277.2_revComp_NCCR_TAg:4894−4998
NC_010277.2_revComp_NCCR_TAg:49−1
NC_010277.2_revComp_NCCR_TAg:490−544
NC_010277.2_revComp_NCCR_TAg:490−565
NC_010277.2_revComp_NCCR_TAg:491−434
NC_010277.2_revComp_NCCR_TAg:4911−4858
NC_010277.2_revComp_NCCR_TAg:4919−4973
NC_010277.2_revComp_NCCR_TAg:4920−5025
NC_010277.2_revComp_NCCR_TAg:4921−5025
NC_010277.2_revComp_NCCR_TAg:4926−4818
NC_010277.2_revComp_NCCR_TAg:4942−4874
NC_010277.2_revComp_NCCR_TAg:4944−5029
NC_010277.2_revComp_NCCR_TAg:4948−4888
NC_010277.2_revComp_NCCR_TAg:4970−5029
NC_010277.2_revComp_NCCR_TAg:4973−4888
NC_010277.2_revComp_NCCR_TAg:4973−4920
NC_010277.2_revComp_NCCR_TAg:4975−4919
NC_010277.2_revComp_NCCR_TAg:4975−5063
NC_010277.2_revComp_NCCR_TAg:4998−4921
NC_010277.2_revComp_NCCR_TAg:5009−5070
NC_010277.2_revComp_NCCR_TAg:5016−5070
NC_010277.2_revComp_NCCR_TAg:5016−5119
NC_010277.2_revComp_NCCR_TAg:5017−5126
NC_010277.2_revComp_NCCR_TAg:5070−5017
NC_010277.2_revComp_NCCR_TAg:5072−5126
NC_010277.2_revComp_NCCR_TAg:5073−5126
NC_010277.2_revComp_NCCR_TAg:5073−5182
NC_010277.2_revComp_NCCR_TAg:509−437
NC_010277.2_revComp_NCCR_TAg:509−458
NC_010277.2_revComp_NCCR_TAg:5106−5215
NC_010277.2_revComp_NCCR_TAg:5107−5216
NC_010277.2_revComp_NCCR_TAg:5109−5163
NC_010277.2_revComp_NCCR_TAg:511−580
NC_010277.2_revComp_NCCR_TAg:5110−5206
NC_010277.2_revComp_NCCR_TAg:5110−5217
NC_010277.2_revComp_NCCR_TAg:5119−5016
NC_010277.2_revComp_NCCR_TAg:5126−5065
NC_010277.2_revComp_NCCR_TAg:5127−5072
NC_010277.2_revComp_NCCR_TAg:5129−5183
NC_010277.2_revComp_NCCR_TAg:5141−5195
NC_010277.2_revComp_NCCR_TAg:5144−5253
NC_010277.2_revComp_NCCR_TAg:5148−5073
NC_010277.2_revComp_NCCR_TAg:5148−5094
NC_010277.2_revComp_NCCR_TAg:5150−5094
NC_010277.2_revComp_NCCR_TAg:5150−5201
NC_010277.2_revComp_NCCR_TAg:5150−5204
NC_010277.2_revComp_NCCR_TAg:5152−5206
NC_010277.2_revComp_NCCR_TAg:5153−5206
NC_010277.2_revComp_NCCR_TAg:5159−5096
NC_010277.2_revComp_NCCR_TAg:5159−5105
NC_010277.2_revComp_NCCR_TAg:5160−5105
NC_010277.2_revComp_NCCR_TAg:5160−5106
NC_010277.2_revComp_NCCR_TAg:5160−5107
NC_010277.2_revComp_NCCR_TAg:5161−5216
NC_010277.2_revComp_NCCR_TAg:5162−5217
NC_010277.2_revComp_NCCR_TAg:5163−5106
NC_010277.2_revComp_NCCR_TAg:5163−5219
NC_010277.2_revComp_NCCR_TAg:5163−5272
NC_010277.2_revComp_NCCR_TAg:5165−5251
NC_010277.2_revComp_NCCR_TAg:5182−5073
NC_010277.2_revComp_NCCR_TAg:5182−5109
NC_010277.2_revComp_NCCR_TAg:5183−5128
NC_010277.2_revComp_NCCR_TAg:5195−5129
NC_010277.2_revComp_NCCR_TAg:5197−5141
NC_010277.2_revComp_NCCR_TAg:5197−5143
NC_010277.2_revComp_NCCR_TAg:5197−5144
NC_010277.2_revComp_NCCR_TAg:5201−5143
NC_010277.2_revComp_NCCR_TAg:5201−5147
NC_010277.2_revComp_NCCR_TAg:5202−5147
NC_010277.2_revComp_NCCR_TAg:5203−5094
NC_010277.2_revComp_NCCR_TAg:5203−5150
NC_010277.2_revComp_NCCR_TAg:5204−5096
NC_010277.2_revComp_NCCR_TAg:5204−5148
NC_010277.2_revComp_NCCR_TAg:5204−5152
NC_010277.2_revComp_NCCR_TAg:5206−5150
NC_010277.2_revComp_NCCR_TAg:5206−5152
NC_010277.2_revComp_NCCR_TAg:5215−5152
NC_010277.2_revComp_NCCR_TAg:5216−5106
NC_010277.2_revComp_NCCR_TAg:5216−5148
NC_010277.2_revComp_NCCR_TAg:5216−5162
NC_010277.2_revComp_NCCR_TAg:5219−5152
NC_010277.2_revComp_NCCR_TAg:5227−5281
NC_010277.2_revComp_NCCR_TAg:5228−5337
NC_010277.2_revComp_NCCR_TAg:5229−5338
NC_010277.2_revComp_NCCR_TAg:5230−5339
NC_010277.2_revComp_NCCR_TAg:5237−5291
NC_010277.2_revComp_NCCR_TAg:5238−5291
NC_010277.2_revComp_NCCR_TAg:524−455
NC_010277.2_revComp_NCCR_TAg:524−470
NC_010277.2_revComp_NCCR_TAg:5251−5142
NC_010277.2_revComp_NCCR_TAg:5253−5197
NC_010277.2_revComp_NCCR_TAg:5256−5150
NC_010277.2_revComp_NCCR_TAg:5256−5199
NC_010277.2_revComp_NCCR_TAg:5258−5162
NC_010277.2_revComp_NCCR_TAg:5258−5202
NC_010277.2_revComp_NCCR_TAg:526−719
NC_010277.2_revComp_NCCR_TAg:5262−5153
NC_010277.2_revComp_NCCR_TAg:5262−5204
NC_010277.2_revComp_NCCR_TAg:5272−5163
NC_010277.2_revComp_NCCR_TAg:5272−5208
NC_010277.2_revComp_NCCR_TAg:5273−5218
NC_010277.2_revComp_NCCR_TAg:5273−5219
NC_010277.2_revComp_NCCR_TAg:5281−5219
NC_010277.2_revComp_NCCR_TAg:5281−5228
NC_010277.2_revComp_NCCR_TAg:5282−5227
NC_010277.2_revComp_NCCR_TAg:5282−5228
NC_010277.2_revComp_NCCR_TAg:5283−5228
NC_010277.2_revComp_NCCR_TAg:5283−5338
NC_010277.2_revComp_NCCR_TAg:5284−5229
NC_010277.2_revComp_NCCR_TAg:5284−5339
NC_010277.2_revComp_NCCR_TAg:5285−5340
NC_010277.2_revComp_NCCR_TAg:5286−5340
NC_010277.2_revComp_NCCR_TAg:5286−5347
NC_010277.2_revComp_NCCR_TAg:5291−5230
NC_010277.2_revComp_NCCR_TAg:5293−5359
NC_010277.2_revComp_NCCR_TAg:5303−5237
NC_010277.2_revComp_NCCR_TAg:5305−5358
NC_010277.2_revComp_NCCR_TAg:5305−5363
NC_010277.2_revComp_NCCR_TAg:5309−5249
NC_010277.2_revComp_NCCR_TAg:5309−5255
NC_010277.2_revComp_NCCR_TAg:5309−5363
NC_010277.2_revComp_NCCR_TAg:5309−5365
NC_010277.2_revComp_NCCR_TAg:5310−5255
NC_010277.2_revComp_NCCR_TAg:5310−5363
NC_010277.2_revComp_NCCR_TAg:5310−5387
NC_010277.2_revComp_NCCR_TAg:5311−5366
NC_010277.2_revComp_NCCR_TAg:5312−5387
NC_010277.2_revComp_NCCR_TAg:5314−5256
NC_010277.2_revComp_NCCR_TAg:5337−5256
NC_010277.2_revComp_NCCR_TAg:5339−5285
NC_010277.2_revComp_NCCR_TAg:5339−5286
NC_010277.2_revComp_NCCR_TAg:5347−5238
NC_010277.2_revComp_NCCR_TAg:5358−5249
NC_010277.2_revComp_NCCR_TAg:5359−5305
NC_010277.2_revComp_NCCR_TAg:5365−5256
NC_010277.2_revComp_NCCR_TAg:5365−5259
NC_010277.2_revComp_NCCR_TAg:5366−5312
NC_010277.2_revComp_NCCR_TAg:542−435
NC_010277.2_revComp_NCCR_TAg:542−470
NC_010277.2_revComp_NCCR_TAg:56−1
NC_010277.2_revComp_NCCR_TAg:580−471
NC_010277.2_revComp_NCCR_TAg:63−122
NC_010277.2_revComp_NCCR_TAg:63−172
NC_010277.2_revComp_NCCR_TAg:64−2
NC_010277.2_revComp_NCCR_TAg:65−10
NC_010277.2_revComp_NCCR_TAg:66−11
NC_010277.2_revComp_NCCR_TAg:66−12
NC_010277.2_revComp_NCCR_TAg:66−13
NC_010277.2_revComp_NCCR_TAg:66−172
NC_010277.2_revComp_NCCR_TAg:665−741
NC_010277.2_revComp_NCCR_TAg:666−774
NC_010277.2_revComp_NCCR_TAg:666−775
NC_010277.2_revComp_NCCR_TAg:68−129
NC_010277.2_revComp_NCCR_TAg:687−749
NC_010277.2_revComp_NCCR_TAg:695−757
NC_010277.2_revComp_NCCR_TAg:698−805
NC_010277.2_revComp_NCCR_TAg:703−765
NC_010277.2_revComp_NCCR_TAg:703−810
NC_010277.2_revComp_NCCR_TAg:711−775
NC_010277.2_revComp_NCCR_TAg:719−665
NC_010277.2_revComp_NCCR_TAg:721−775
NC_010277.2_revComp_NCCR_TAg:721−797
NC_010277.2_revComp_NCCR_TAg:73−12
NC_010277.2_revComp_NCCR_TAg:740−797
NC_010277.2_revComp_NCCR_TAg:749−695
NC_010277.2_revComp_NCCR_TAg:75−128
NC_010277.2_revComp_NCCR_TAg:75−141
NC_010277.2_revComp_NCCR_TAg:756−813
NC_010277.2_revComp_NCCR_TAg:765−698
NC_010277.2_revComp_NCCR_TAg:765−711
NC_010277.2_revComp_NCCR_TAg:767−861
NC_010277.2_revComp_NCCR_TAg:774−665
NC_010277.2_revComp_NCCR_TAg:794−687
NC_010277.2_revComp_NCCR_TAg:805−743
NC_010277.2_revComp_NCCR_TAg:807−861
NC_010277.2_revComp_NCCR_TAg:807−862
NC_010277.2_revComp_NCCR_TAg:808−917
NC_010277.2_revComp_NCCR_TAg:810−740
NC_010277.2_revComp_NCCR_TAg:813−751
NC_010277.2_revComp_NCCR_TAg:821−714
NC_010277.2_revComp_NCCR_TAg:821−759
NC_010277.2_revComp_NCCR_TAg:828−886
NC_010277.2_revComp_NCCR_TAg:832−938
NC_010277.2_revComp_NCCR_TAg:85−19
NC_010277.2_revComp_NCCR_TAg:859−913
NC_010277.2_revComp_NCCR_TAg:861−913
NC_010277.2_revComp_NCCR_TAg:861−969
NC_010277.2_revComp_NCCR_TAg:87−141
NC_010277.2_revComp_NCCR_TAg:87−171
NC_010277.2_revComp_NCCR_TAg:882−808
NC_010277.2_revComp_NCCR_TAg:882−936
NC_010277.2_revComp_NCCR_TAg:883−992
NC_010277.2_revComp_NCCR_TAg:884−969
NC_010277.2_revComp_NCCR_TAg:9−110
NC_010277.2_revComp_NCCR_TAg:913−828
NC_010277.2_revComp_NCCR_TAg:915−992
NC_010277.2_revComp_NCCR_TAg:917−859
NC_010277.2_revComp_NCCR_TAg:918−863
NC_010277.2_revComp_NCCR_TAg:918−864
NC_010277.2_revComp_NCCR_TAg:936−864
NC_010277.2_revComp_NCCR_TAg:937−882
NC_010277.2_revComp_NCCR_TAg:938−883
NC_010277.2_revComp_NCCR_TAg:938−993
NC_010277.2_revComp_NCCR_TAg:939−1100
NC_010277.2_revComp_NCCR_TAg:939−993
NC_010277.2_revComp_NCCR_TAg:992−883
NA
V3
1e+05
2e+05
3e+05
4e+05

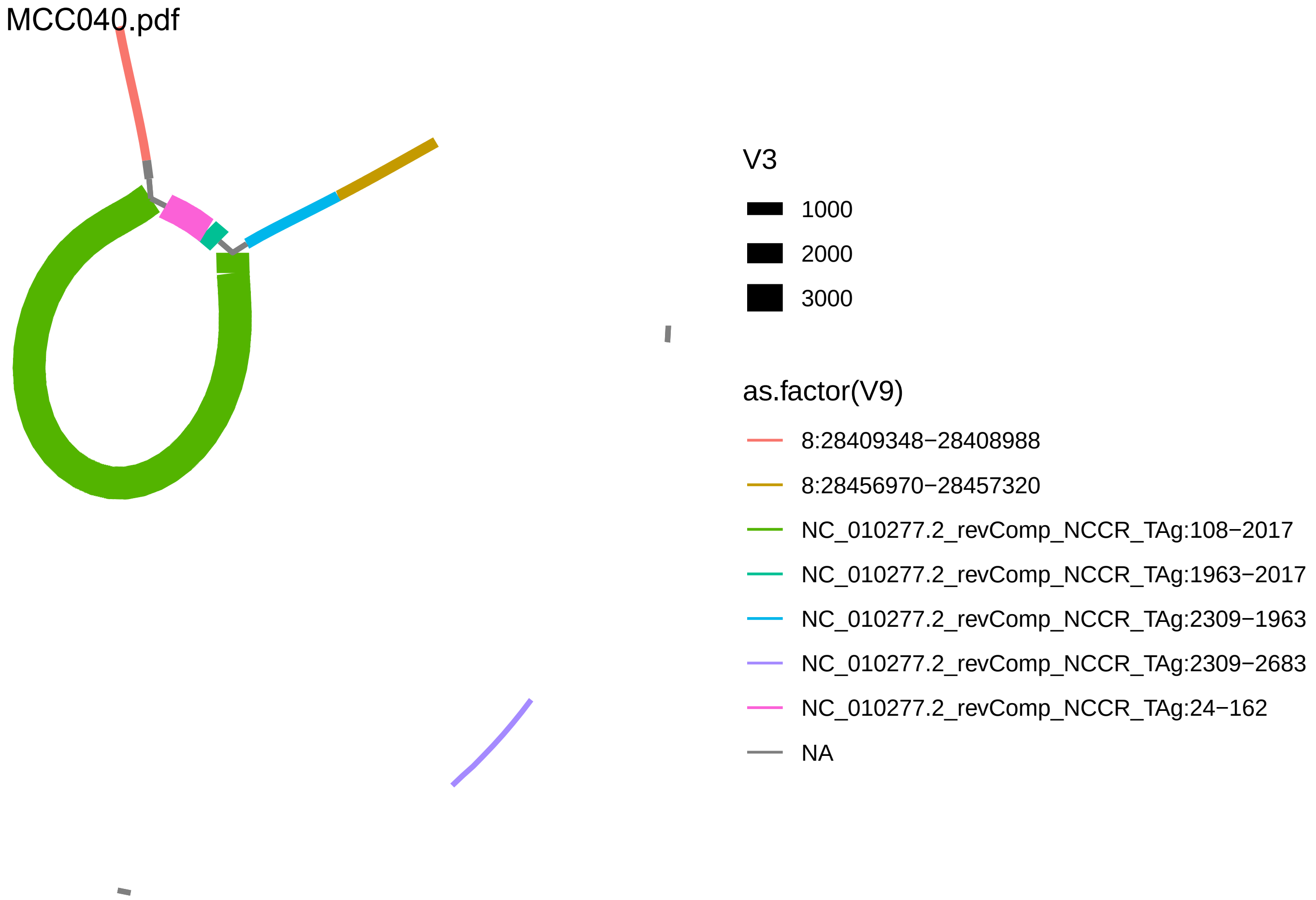
MCC040.pdf
V3
1000
2000
3000
as.factor(V9)
8:28409348−28408988
8:28456970−28457320
NC_010277.2_revComp_NCCR_TAg:108−2017
NC_010277.2_revComp_NCCR_TAg:1963−2017
NC_010277.2_revComp_NCCR_TAg:2309−1963
NC_010277.2_revComp_NCCR_TAg:2309−2683
NC_010277.2_revComp_NCCR_TAg:24−162
NA

MCC041.pdf

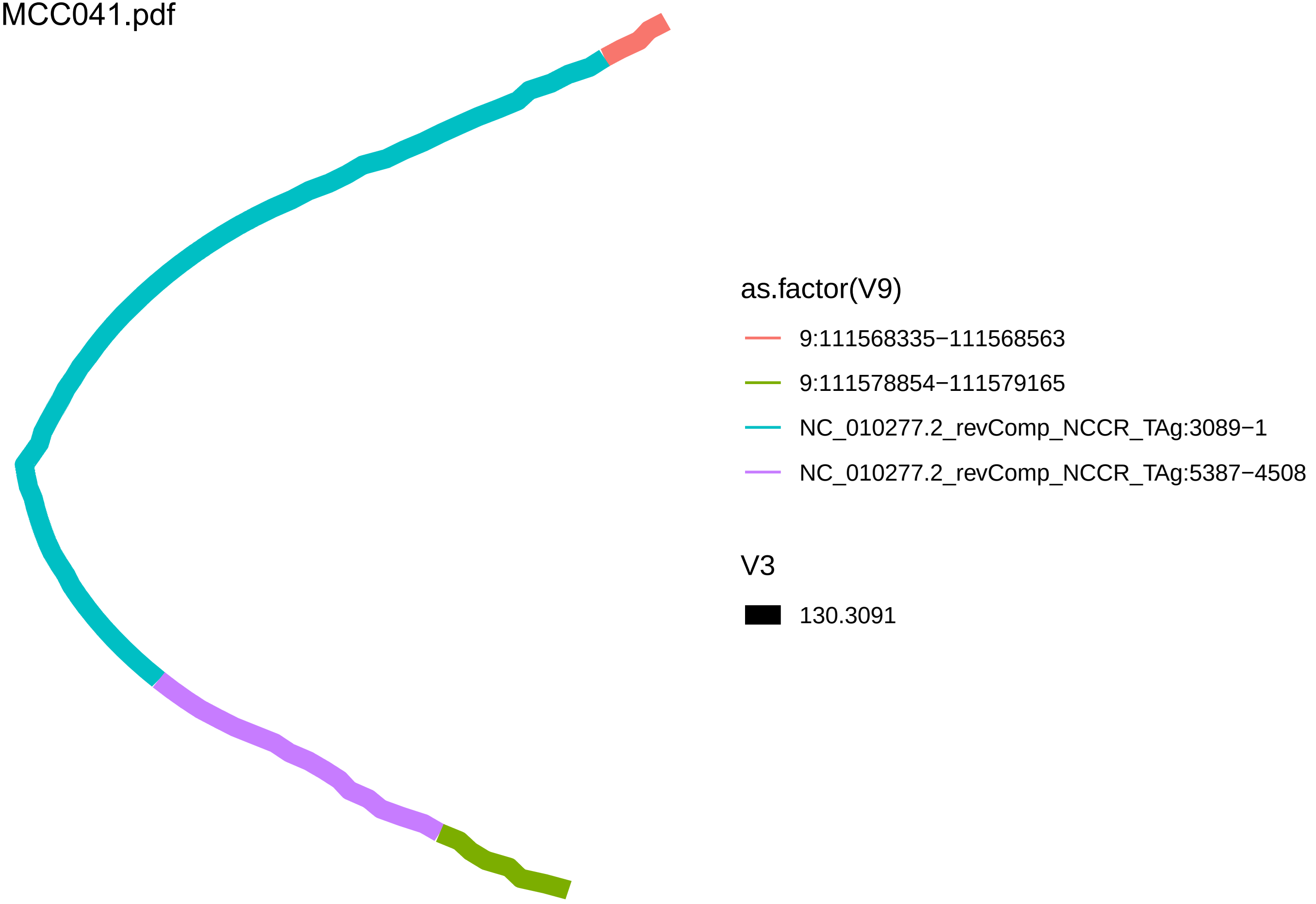
as.factor(V9)
9:111568335−111568563
9:111578854−111579165
NC_010277.2_revComp_NCCR_TAg:3089−1
NC_010277.2_revComp_NCCR_TAg:5387−4508
V3
130.3091

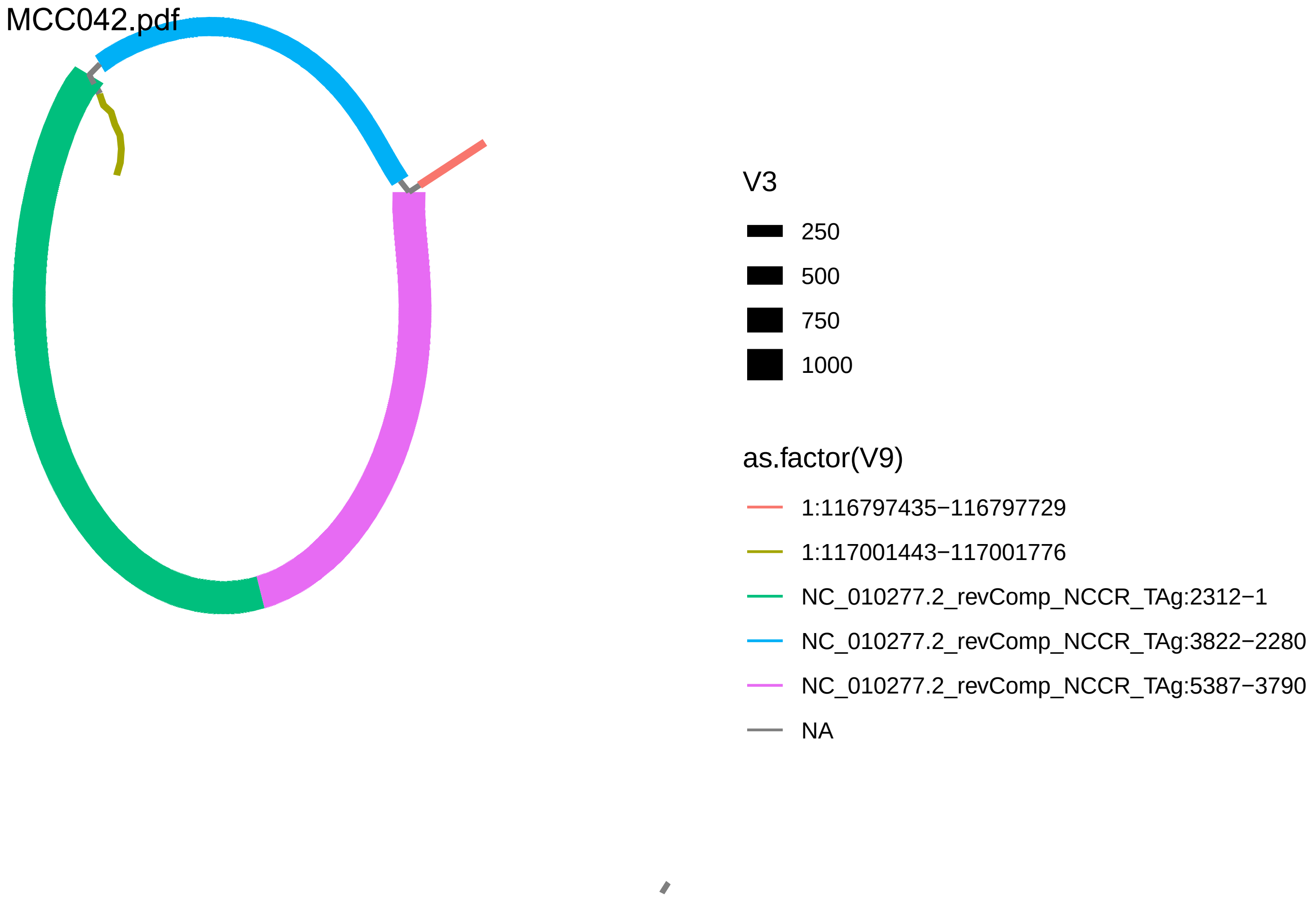

MCC042.pdf
V3
250
500
750
1000
as.factor(V9)
1:116797435−116797729
1:117001443−117001776
NC_010277.2_revComp_NCCR_TAg:2312−1
NC_010277.2_revComp_NCCR_TAg:3822−2280
NC_010277.2_revComp_NCCR_TAg:5387−3790
NA

MCC043.pdf

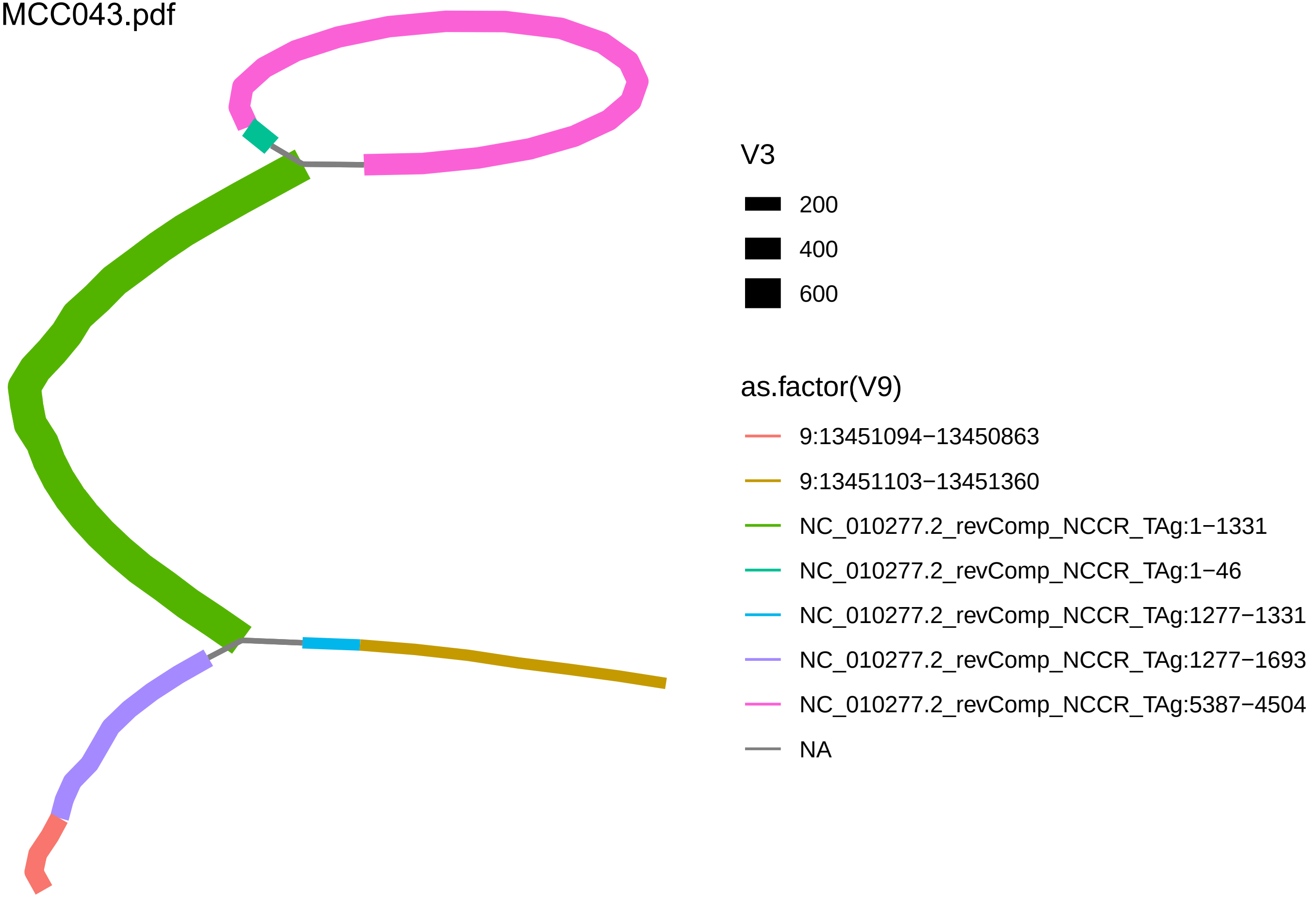

V3
200
400
600
as.factor(V9)
9:13451094−13450863
9:13451103−13451360
NC_010277.2_revComp_NCCR_TAg:1−1331
NC_010277.2_revComp_NCCR_TAg:1−46
NC_010277.2_revComp_NCCR_TAg:1277−1331
NC_010277.2_revComp_NCCR_TAg:1277−1693
NC_010277.2_revComp_NCCR_TAg:5387−4504
NA

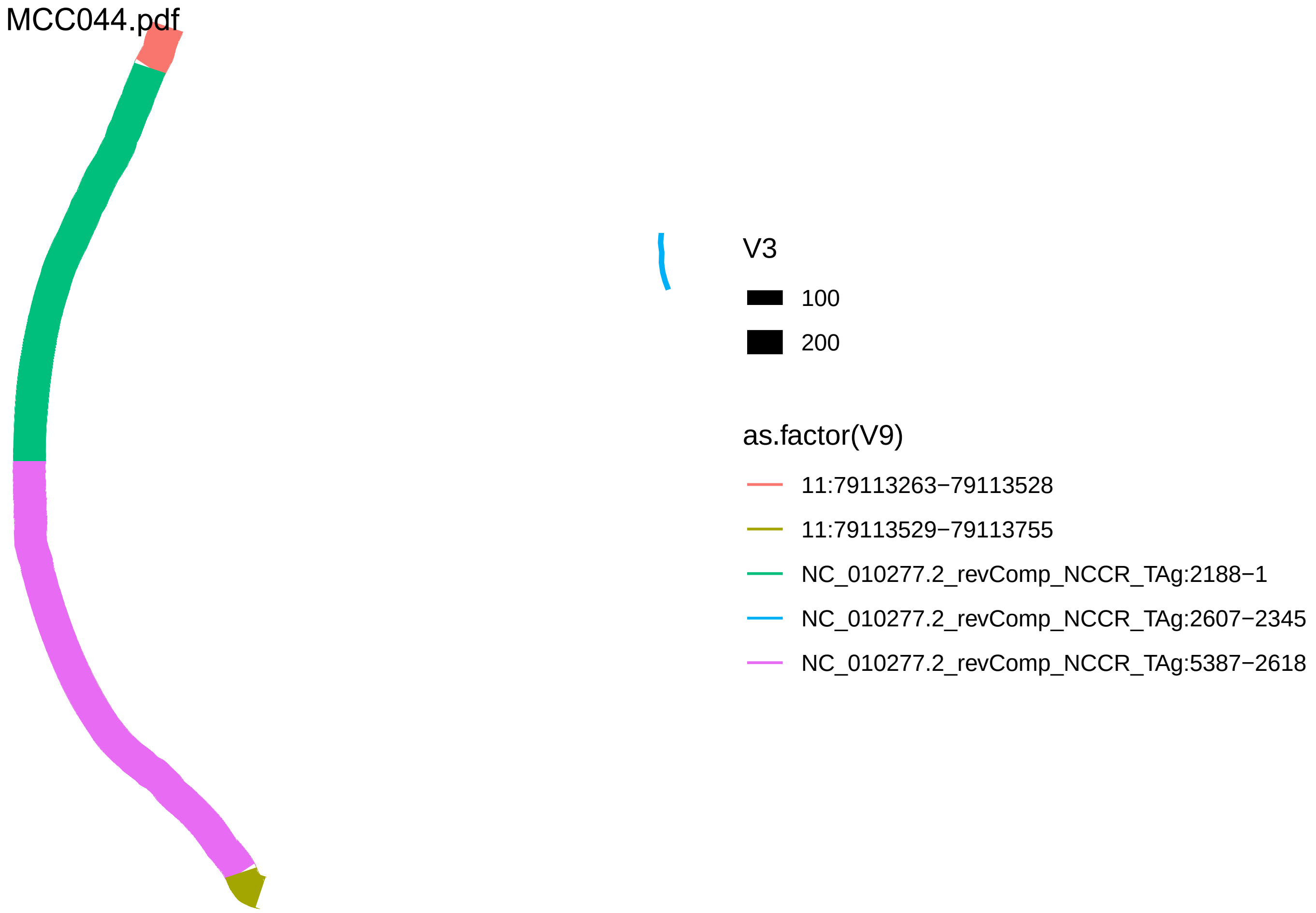
MCC044.pdf
V3
100
200
as.factor(V9)
11:79113263−79113528
11:79113529−79113755
NC_010277.2_revComp_NCCR_TAg:2188−1
NC_010277.2_revComp_NCCR_TAg:2607−2345
NC_010277.2_revComp_NCCR_TAg:5387−2618

MCC047.pdf

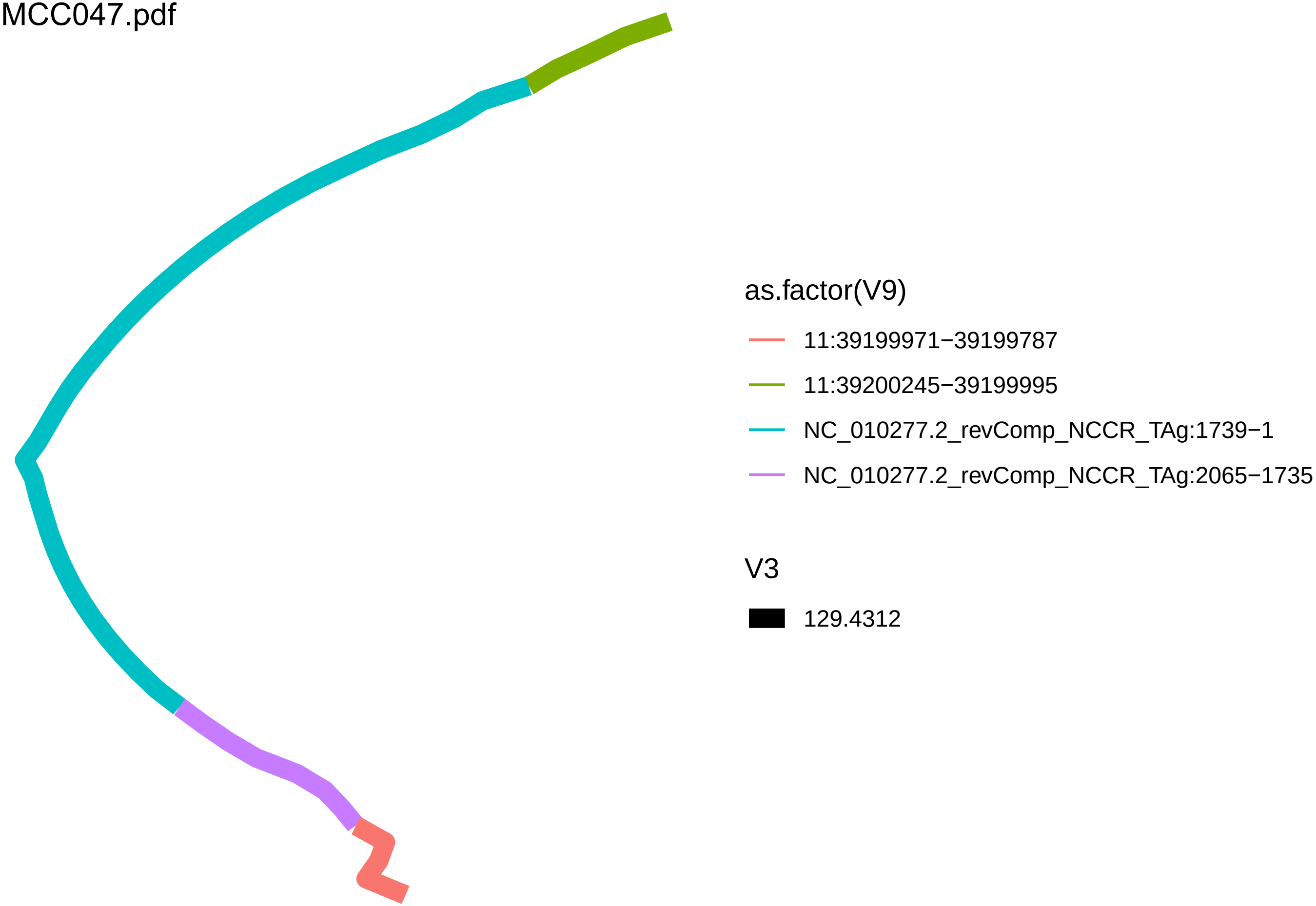

as.factor(V9)
11:39199971−39199787
11:39200245−39199995
NC_010277.2_revComp_NCCR_TAg:1739−1
NC_010277.2_revComp_NCCR_TAg:2065−1735
V3
129.4312

MCC050.pdf

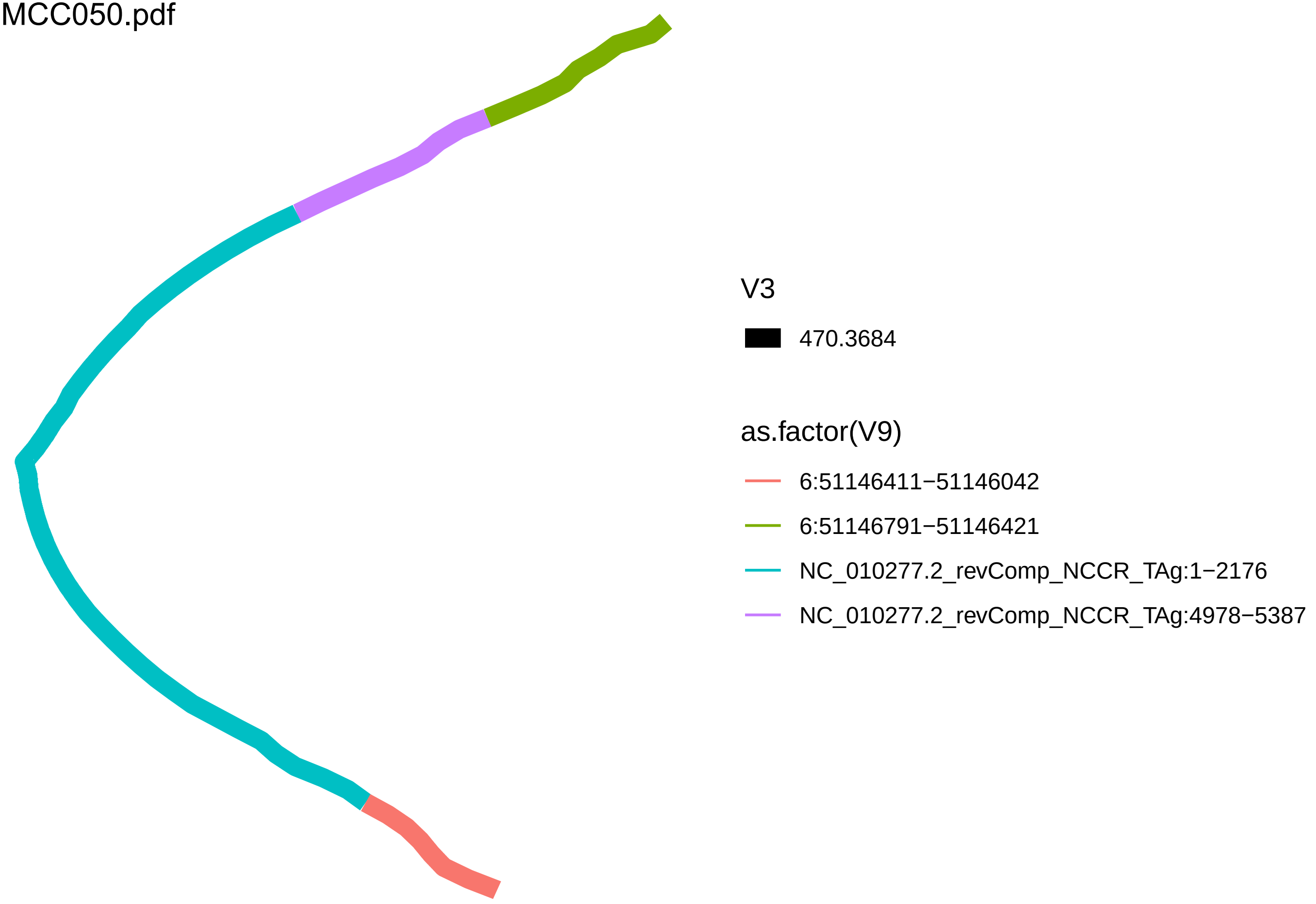
V3
470.3684
as.factor(V9)
6:51146411−51146042
6:51146791−51146421
NC_010277.2_revComp_NCCR_TAg:1−2176
NC_010277.2_revComp_NCCR_TAg:4978−5387

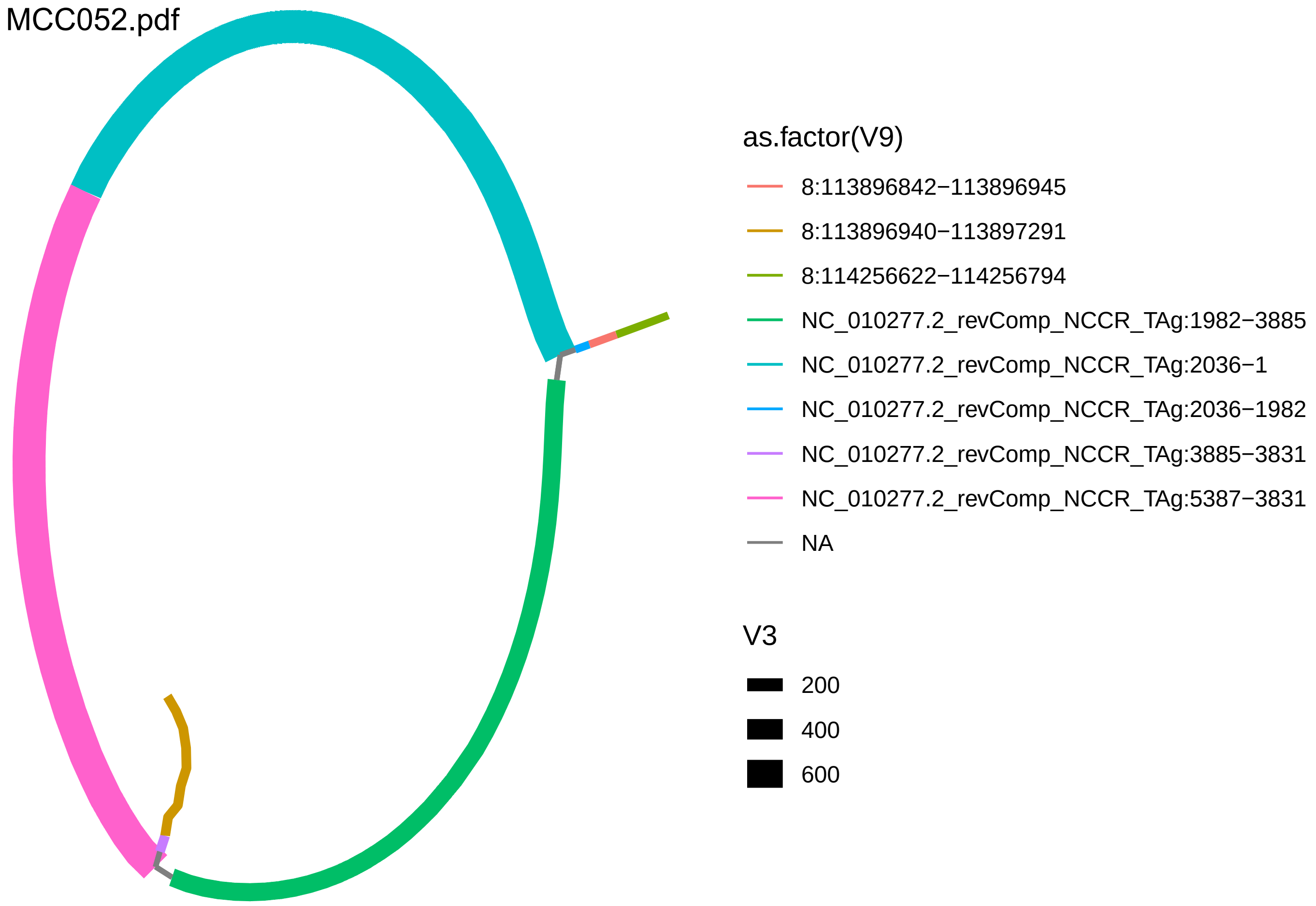
MCC052.pdf
as.factor(V9)
8:113896842−113896945
8:113896940−113897291
8:114256622−114256794
NC_010277.2_revComp_NCCR_TAg:1982−3885
NC_010277.2_revComp_NCCR_TAg:2036−1
NC_010277.2_revComp_NCCR_TAg:2036−1982
NC_010277.2_revComp_NCCR_TAg:3885−3831
NC_010277.2_revComp_NCCR_TAg:5387−3831
NA
V3
200
400
600

MCC054.pdf

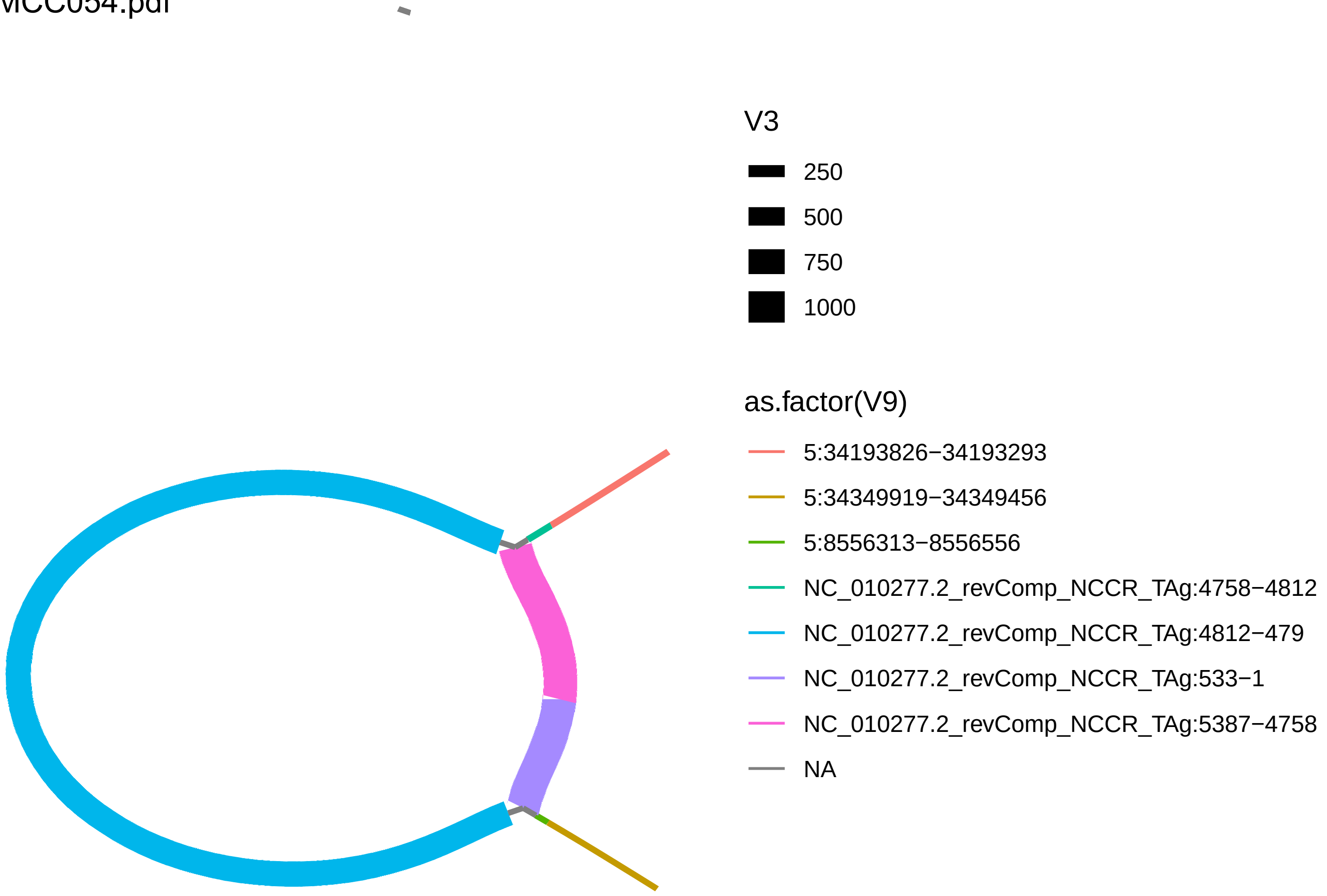

V3
250
500
750
1000
as.factor(V9)
5:34193826−34193293
5:34349919−34349456
5:8556313−8556556
NC_010277.2_revComp_NCCR_TAg:4758−4812
NC_010277.2_revComp_NCCR_TAg:4812−479
NC_010277.2_revComp_NCCR_TAg:533−1
NC_010277.2_revComp_NCCR_TAg:5387−4758
NA

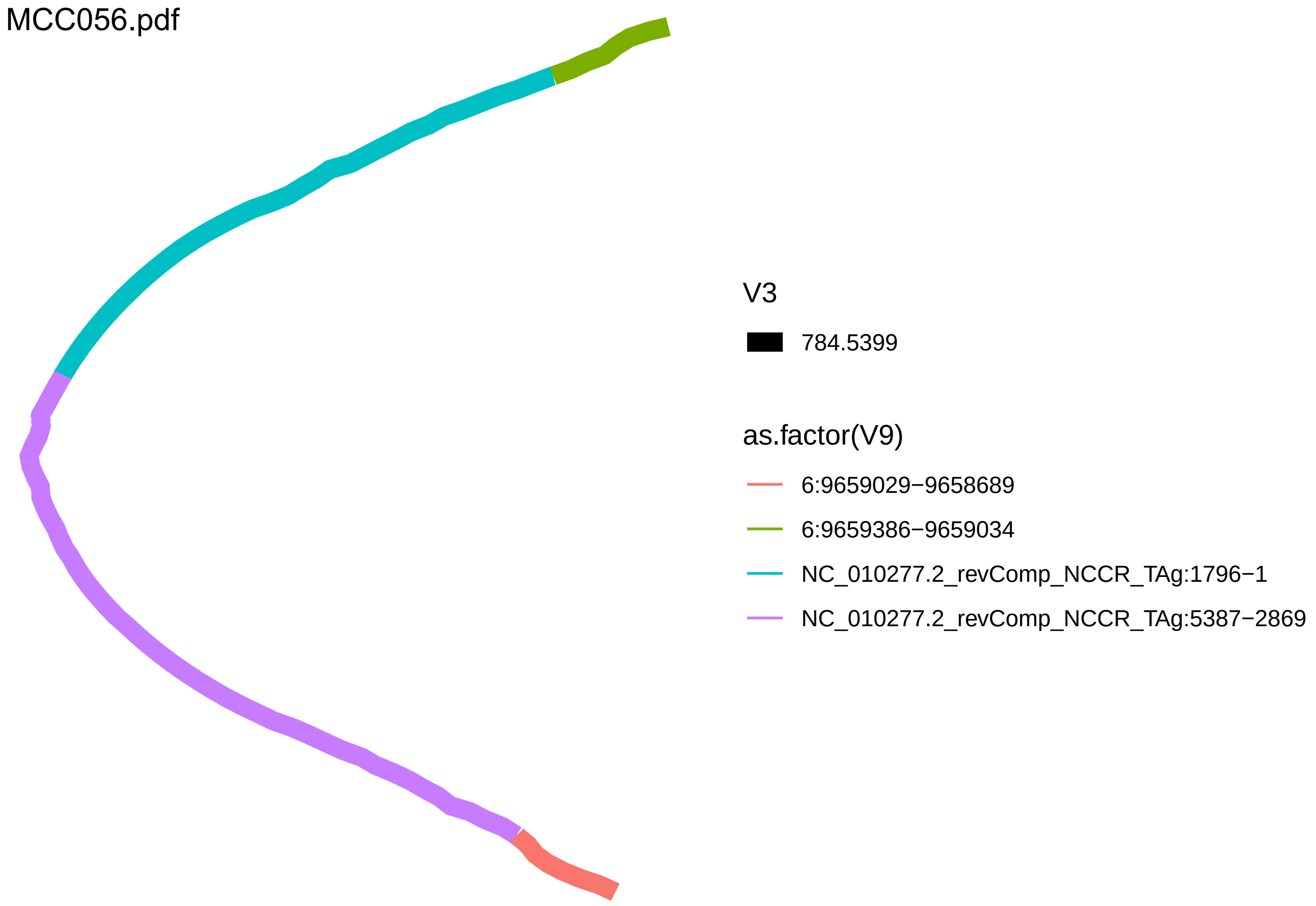

MCC056.pdf
V3
784.5399
as.factor(V9)
6:9659029−9658689
6:9659386−9659034
NC_010277.2_revComp_NCCR_TAg:1796−1
NC_010277.2_revComp_NCCR_TAg:5387−2869

MCC062.pdf

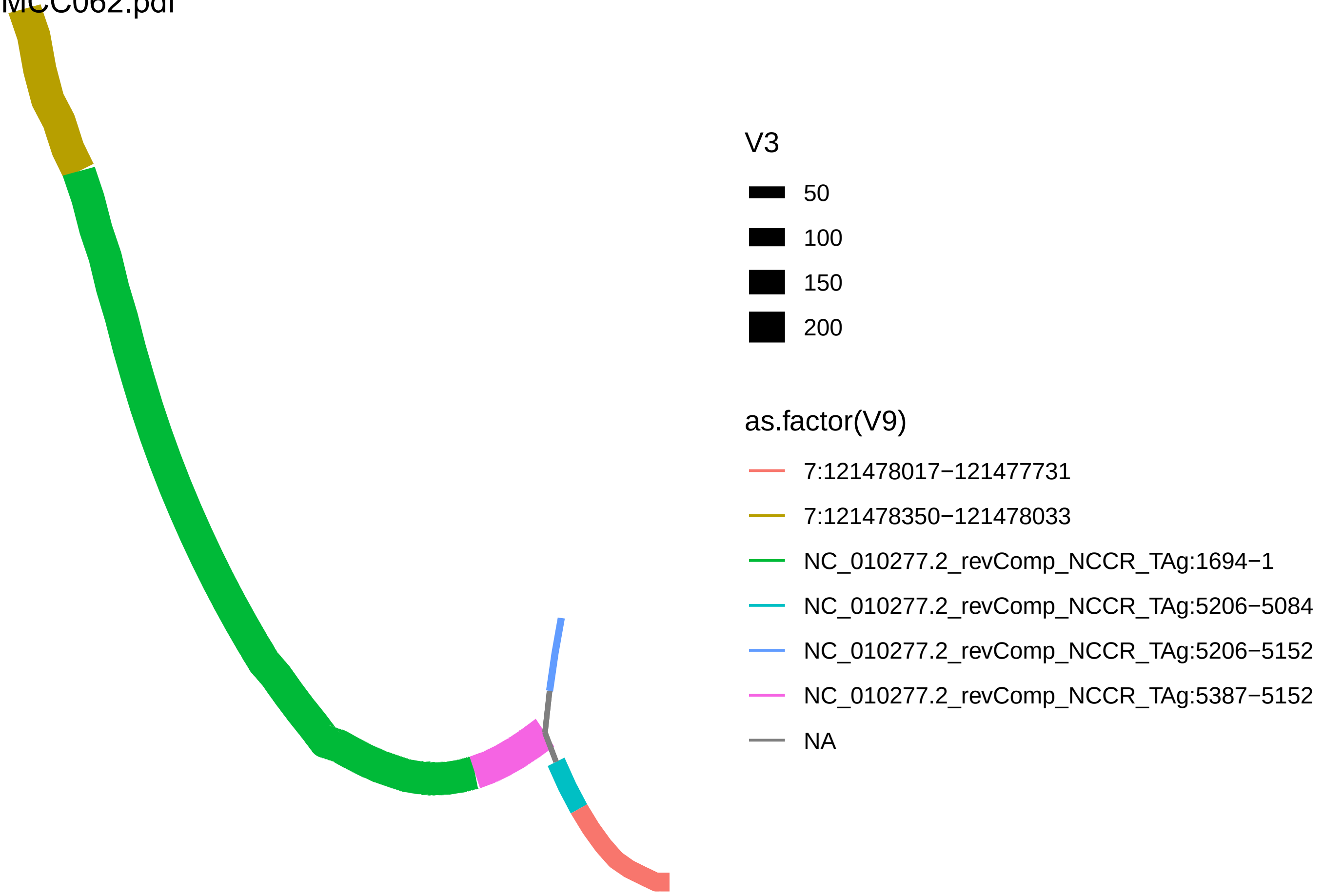

V3
50
100
150
200
as.factor(V9)
7:121478017−121477731
7:121478350−121478033
NC_010277.2_revComp_NCCR_TAg:1694−1
NC_010277.2_revComp_NCCR_TAg:5206−5084
NC_010277.2_revComp_NCCR_TAg:5206−5152
NC_010277.2_revComp_NCCR_TAg:5387−5152
NA

MCC069.pdf

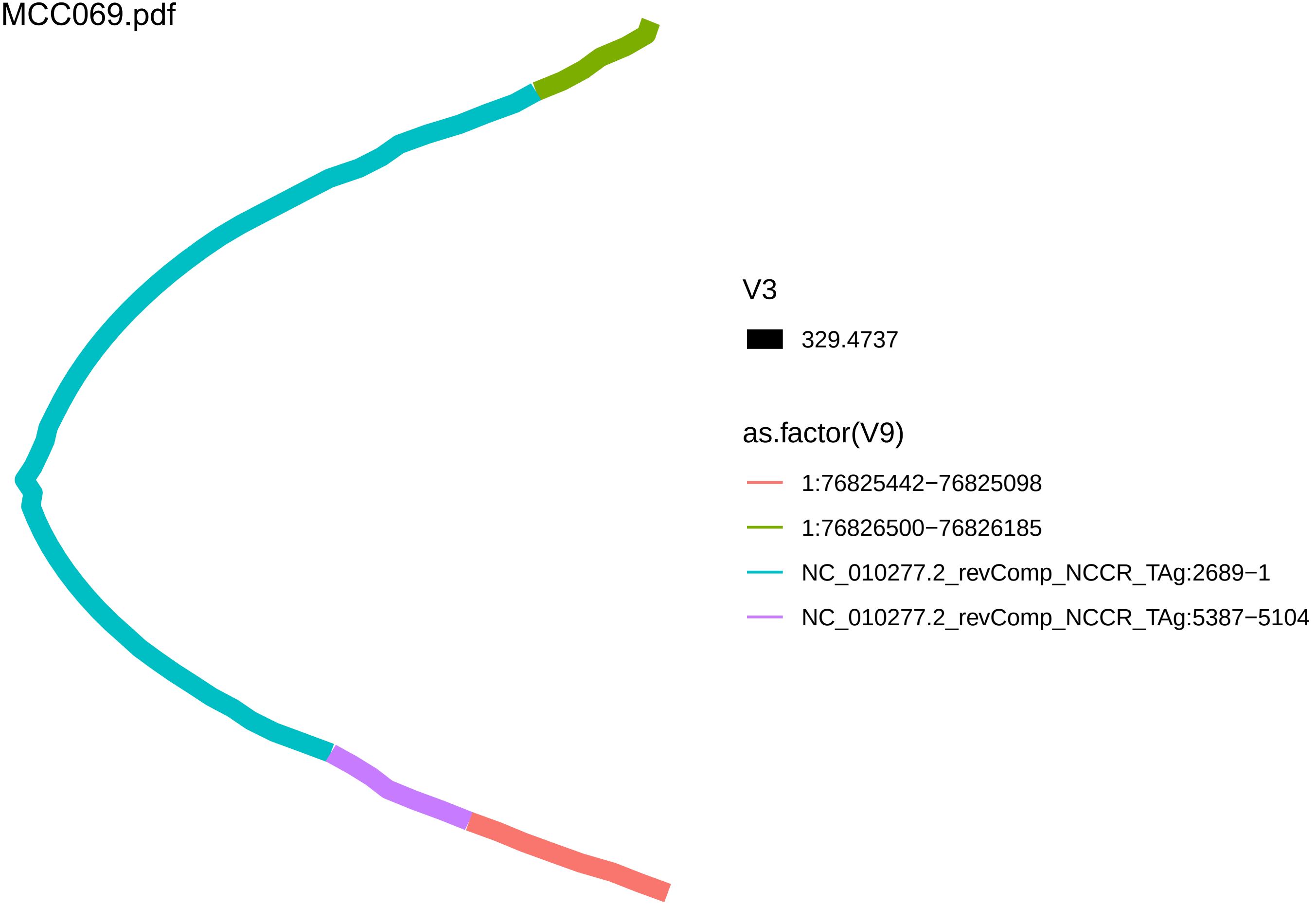
V3
329.4737
as.factor(V9)
1:76825442−76825098
1:76826500−76826185
NC_010277.2_revComp_NCCR_TAg:2689−1
NC_010277.2_revComp_NCCR_TAg:5387−5104

MCC071.pdf

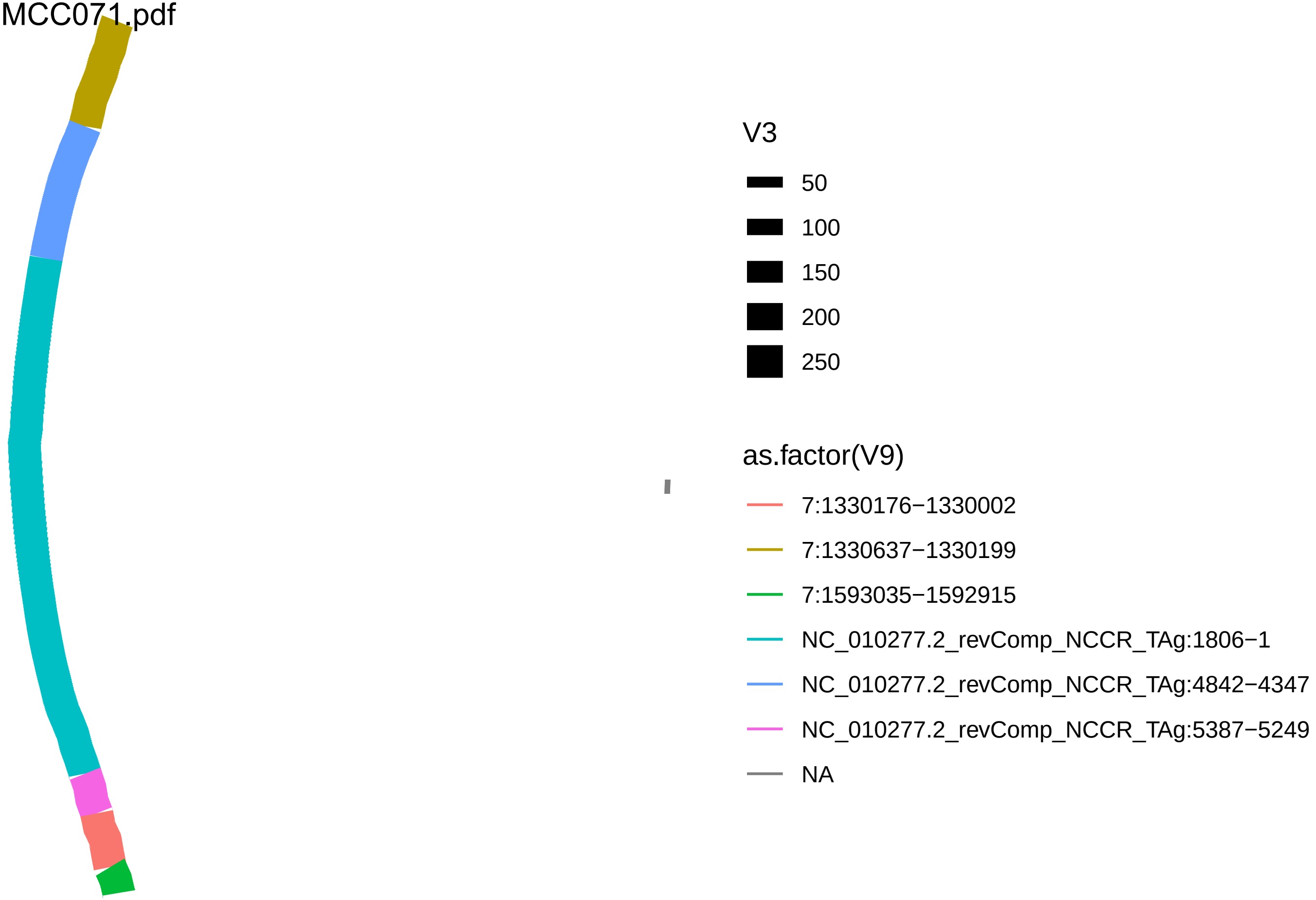
V3
50
100
150
200
250
as.factor(V9)
7:1330176−1330002
7:1330637−1330199
7:1593035−1592915
NC_010277.2_revComp_NCCR_TAg:1806−1
NC_010277.2_revComp_NCCR_TAg:4842−4347
NC_010277.2_revComp_NCCR_TAg:5387−5249
NA
